# Combating pan-coronavirus infection by indomethacin through simultaneously inhibiting viral replication and inflammatory response

**DOI:** 10.1016/j.isci.2023.107631

**Published:** 2023-08-16

**Authors:** Yining Wang, Pengfei Li, Lei Xu, Annemarie C. de Vries, Robbert J. Rottier, Wenshi Wang, Marie-Rose B.S. Crombag, Maikel P. Peppelenbosch, Denis E. Kainov, Qiuwei Pan

**Affiliations:** 1 Department of Gastroenterology and Hepatology, Erasmus MC-University Medical Center, Rotterdam, the Netherlands; 2 State Key Laboratory of Crop Stress Biology for Arid Areas, Shaanxi Key Laboratory of Agricultural and Environmental Microbiology, College of Life Sciences, Northwest A&F University, Yangling 712100, Shaanxi, China; 3 Department of Pediatric Surgery, Erasmus MC-Sophia Children’s Hospital, Rotterdam, the Netherlands; 4 Department of Cell Biology, Erasmus MC-University Medical Center, Rotterdam, the Netherlands; 5 Jiangsu Key Laboratory of Immunity and Metabolism, Department of Pathogenic Biology and Immunology, Xuzhou Medical University, Xuzhou, China; 6 Department of Hospital Pharmacy, Erasmus MC-University Medical Center, Rotterdam, the Netherlands; 7 Department of Clinical and Molecular Medicine, Norwegian University of Science and Technology, 7028 Trondheim, Norway; 8 Institute of Technology, University of Tartu, 50090 Tartu, Estonia

**Keywords:** Drugs, Immune response, Virology

## Abstract

Severe infections with coronaviruses are often accompanied with hyperinflammation, requiring therapeutic strategies to simultaneously tackle the virus and inflammation. By screening a safe-in-human broad-spectrum antiviral agents library, we identified that indomethacin can inhibit pan-coronavirus infection in human cell and airway organoids models. Combining indomethacin with oral antiviral drugs authorized for treating COVID-19 results in synergistic anti-coronavirus activity. Coincidentally, screening a library of FDA-approved drugs identified indomethacin as the most potent potentiator of interferon response through increasing STAT1 phosphorylation. Combining indomethacin with interferon-alpha exerted synergistic antiviral effects against multiple coronaviruses. The anti-coronavirus activity of indomethacin is associated with activating interferon response. In a co-culture system of lung epithelial cells with macrophages, indomethacin inhibited both viral replication and inflammatory response. Collectively, indomethacin is a pan-coronavirus inhibitor that can simultaneously inhibit virus-triggered inflammatory response. The therapeutic potential of indomethacin can be further augmented by combining it with oral antiviral drugs or interferon-alpha.

## Highlights

• Indomethacin exerts pan-coronavirus antiviral activity
• Indomethacin promotes interferon response through enhancing STAT1 phosphorylation
• Indomethacin inhibits coronavirus-triggered inflammatory response
• Augmenting indomethacin by combining with oral antiviral drugs or interferon-alpha

## Graphical abstract

**Figure:**
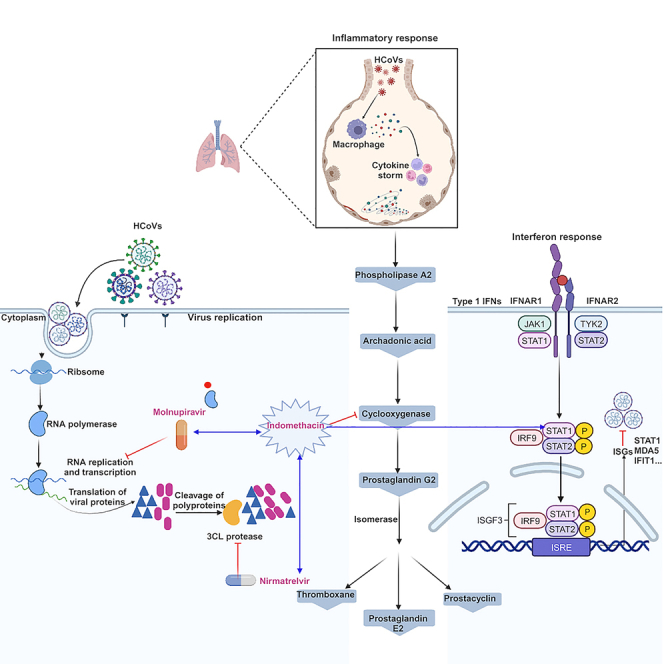

## Introduction

Coronaviruses are a large family of single-stranded positive-sense RNA viruses circulating among various natural hosts. There are seven types of coronaviruses known to infect humans—four seasonal coronaviruses (229E, OC43, NL63, and HKU1) and three highly pathogenic coronaviruses (SARS-CoV, MERS-CoV, and SARS-CoV-2).^1^ SARS-CoV-2, infecting both the upper and lower respiratory tract, has caused over 6 million deaths worldwide until May 2023, but this virus is continuously evolving showing a clear trend toward less pathogenic, in particular the Omicron variant.^2^^,^^3^^,^^4^ Seasonal coronaviruses primarily infect the upper respiratory tract and only cause the common cold. However, severe complications including death can occur especially in vulnerable populations such as children, elderly people, and immunocompromised patients.^5^^,^^6^

Several virus-targeted oral drugs including Paxlovid (nirmatrelvir as the main active component) and molnupiravir have been approved for treating COVID-19 caused by SARS-CoV-2 infection.^7^ Repurposing these COVID-19 medications represents an attractive approach for treating seasonal coronavirus infections. However, this approach is not always straightforward. For example, we recently found that nirmatrelvir has minimum antiviral activity against NL63 infection.^8^ Thus, there is a need to identify pan-coronavirus inhibitors that are effective against different circulating coronaviruses, which can also serve as preparedness for future newly surfaced coronaviruses or variants.

Antiviral monotherapies are often found to be suboptimal in clinical settings with respect to efficacy as well as risks of drug resistance development.^9^ For example, COVID-19 rebound has been reported in a substantial number of patients following the cessation of Paxlovid or molnupiravir antiviral treatment.^10^^,^^11^ Although the mechanisms of rebound remain unclear, it is suspected to be associated with the development of drug resistance. In contrast, combinational antiviral therapies can exhibit synergism and prevent the development of drug-resistant strains by completely halting viral replication.^9^ It is a common and effective strategy to combine antiviral drugs with complementary mechanisms-of-action, for example combining virus-targeted with host-targeted agents. The host interferon (IFN) pathway acts as a first-line defense against viral infections.^12^ Thus, the recombinant IFN-α has been widely used as a broad-spectrum antiviral drug for treating various viral diseases in the clinic.^13^ Combinational treatment of IFN-α and other antiviral drugs has been extensively explored for potentiating therapeutic efficacy and preventing drug resistance development in clinical practice.^14^^,^^15^ Interestingly, as an exception, it has been shown that IFN-α treatment can promote the infection of seasonal coronavirus OC43 possible through the IFN-inducible transmembrane (IFITM) proteins to facilitate its entry into host cells.^16^^,^^17^

Inflammatory response is a protective mechanism involving immune cells to eliminate the initial cause of cell injury and clear out necrotic cells and tissues damaged from the original insult such as the virus. But inflammatory response must be timely terminated when no longer needed to prevent unnecessary “bystander” damage to tissues. Unfortunately, this essential feedback mechanism is likely dysregulated during severe acute viral infections, thus resulting in pathological inflammation. Massive inflammation accompanied with a cytokine storm is a prominent feature for severe COVID-19 patients, contributing to morbidity and mortality.^18^^,^^19^ For these patients, we postulate that antiviral therapy alone is insufficient but further combination with anti-inflammatory treatment may be needed to achieve improved outcomes.

In this study, through screening a library of safe-in-human broad-spectrum antiviral agents, we identified indomethacin, a traditional nonsteroidal anti-inflammatory drug (NSAID), as a pan-coronavirus inhibitor. Combination of indomethacin with the COVID-19 antiviral drugs resulted in enhanced antiviral activity. Interestingly, we found that indomethacin can augment host interferon response but inhibit coronavirus-triggered inflammatory response. Finally, we demonstrated that indomethacin simultaneously inhibited viral replication and coronavirus-induced inflammatory response in a co-culture system of human lung epithelial cells with macrophages.

## Results

### Screening a broad-spectrum antiviral drug library identifies indomethacin as a pan-coronavirus inhibitor

**Figure 1:**
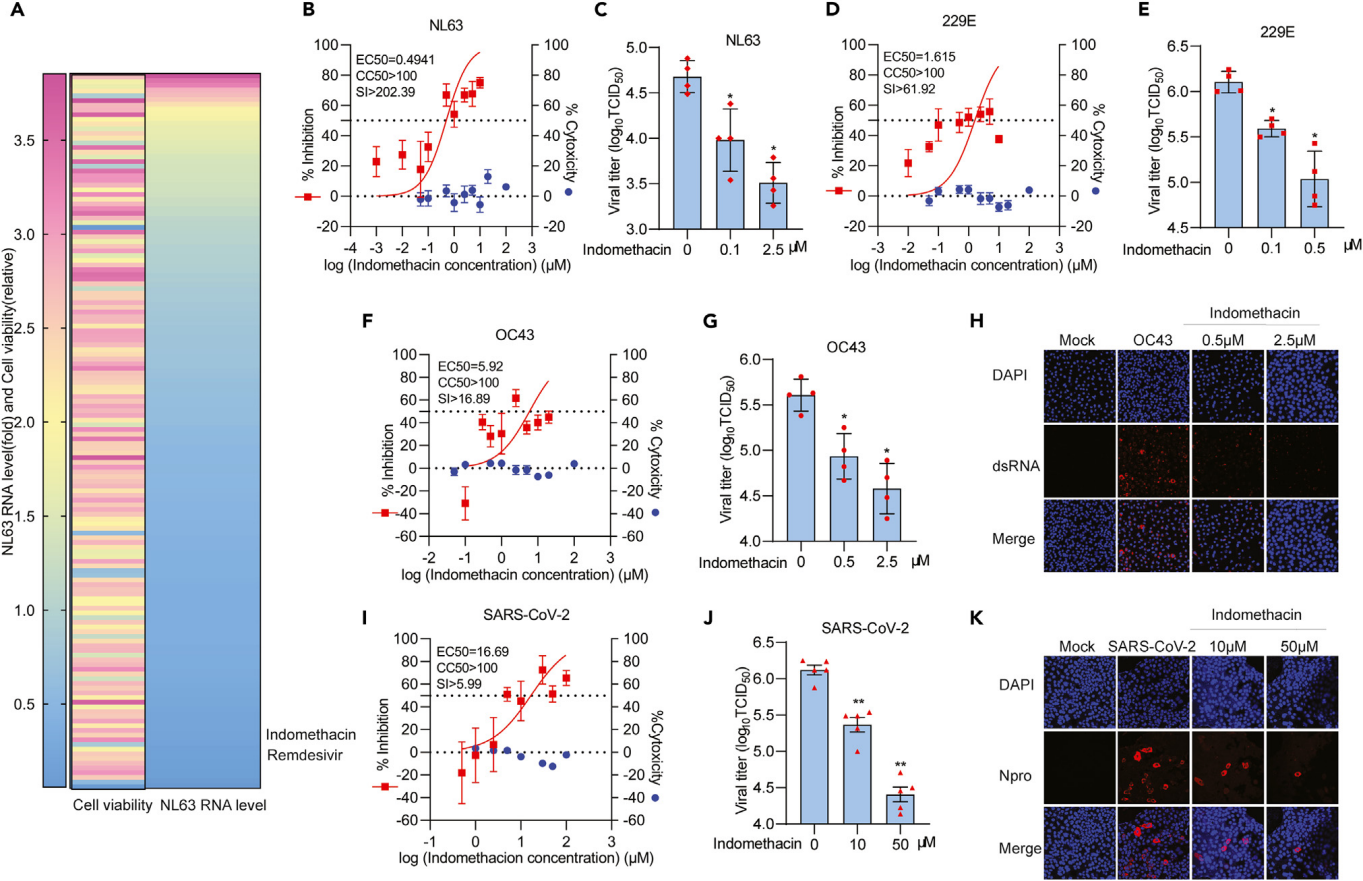
Drug screening identified indomethacin as an inhibitor of pan-coronavirus infection (A) Caco-2 cell line was infected with NL63 at a multiplicity of infection (MOI) of 0.1 overnight and treated antiviral agents at 2.5 μM for 48 h. Cell viability was determined by MTT. (B, D, F, and I) Caco-2, A549 or Calu-3 cells were infected with NL63, 229E, OC43 or SARS-CoV-2 at different MOI in the treatment of different concentrations of indomethacin for 48 h. Viral yield in cells was quantified by qRT-PCR. Cytotoxicity was determined by MTT assay. The EC50 and CC50 values were presented. The left and right Y axis of the graphs represent mean % inhibition of virus yield and cytotoxicity of the drugs, respectively. (n = 5–20). (C, E, G, and J) TCID50 assay quantifying titers of secreted infectious NL63, 229E, OC43 or SARS-CoV-2 virus particles at 48 h post-treatment of different concentrations of indomethacin (n = 4–5). (H and K) Immunofluorescence staining of dsRNA or Npro of SARS-CoV-2 in OC43 infected A549 or SARS-CoV-2 infected Calu-3 cells treated with or without different concentrations of indomethacin. DAPI (blue) was applied to visualize nuclei. (Scale bar, 100 μm. 40x oil immersion objective). Data represent as mean ± SEM. ∗p < 0.05; ∗∗p < 0.01; ∗∗∗p < 0.001. (Mann-Whitney test) See also Figure S1, Tables S1 and S3.

To identify potential anti-coronavirus candidates, we screened a safe-in-human broad-spectrum antiviral agents library consisting of about 150 drugs/compounds (Table S1) in human intestinal Caco-2 cells infected with NL63 (Figure 1A). NL63 is the only member of seasonal coronaviruses that utilizes angiotensin-converting enzyme 2 (ACE2) as its receptor for viral entry,^20^ which is similar to SARS-CoV and SARS-CoV-2. To minimize nonspecific effects on host cells, we used a relatively low concentration of 2.5 μM or DMSO vehicle control and treated for 48 h. By qRT-PCR (primers listed in Table S2) quantification of NL63 genomic RNA, we identified 22 candidates exerting over 70% inhibitory effects and less than 50% cytotoxicity (Figure 1A and Table S3). The antiviral activities of these candidates are equivalent to or more potent than that of the positive control, remdesivir. Among these, the widely used NSAID drug indomethacin showed strong anti-NL63 activity. Severe infections with coronaviruses are often accompanied with hyperinflammation, which drives morbidity and mortality.^18^^,^^19^ Considering such a drug candidate may simultaneously inhibit viral replication and pathological inflammation, we thus prioritized indomethacin for further detailed study.

We next profiled a series of concentrations (0.001–10 μM) and revealed a dose-dependent antiviral activity. We observed a large therapeutic window between cytotoxic and antiviral activities, as shown that the half maximum cytotoxic concentration (CC50) value was over 100 μM, half maximum effective concentration (EC50) value was 0.4941 μM, and selectivity index (SI) was over 200 (Figure 1B). Consistently, the titers of produced NL63 virus with infectivity were significantly reduced by indomethacin treatment, determined by TCID50 assay using harvested supernatant of infected Caco-2 cells at 48 h post-treatment (Figure 1C). Immunofluorescent staining of the viral double-strand RNA (dsRNA) also showed a dramatic reduction in the number of infected cells by indomethacin treatment (Figure S1A). Similar results were observed in monkey LLCMK-2 cell line which is widely used for propagating the NL63 virus in laboratory and human liver Huh7 cell line (Figures S1B–S1E). For example, treatment of 5 μM indomethacin resulted in over 50% reduction of viral RNA level with mild effect on cell viability (Figures S1B–S1E).

**Figure 2:**
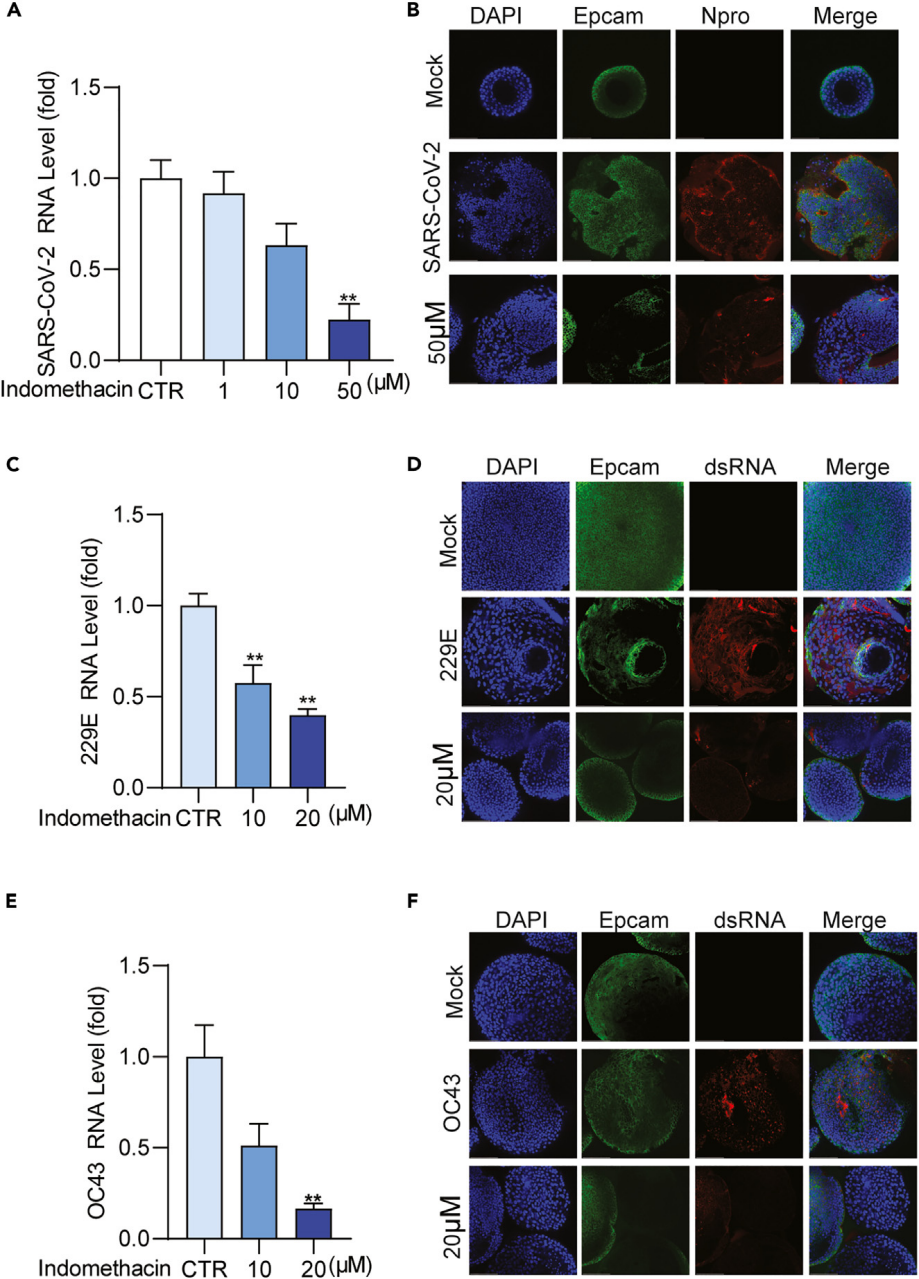
Indomethacin against pan-coronavirus replication in human airway organoids models hAOs were infected with SARS-CoV-2, 229E, or OC43 at different MOI in the treatment of different concentrations of indomethacin for 48 h. (A, C, and E) The effects of indomethacin on intracellular viral RNA levels of SARS-CoV-2, 229E or OC43 in hAOs (n = 5). (B, D, and F) Immunofluorescence analysis of Npro of SARS-CoV-2 or viral dsRNA in hAOs treated with different concentrations of indomethacin for 48 h. DAPI (blue) and Epcam (green) were applied to visualize nuclei and cytomembrane. (Scale bar, 100 μm. 40x oil immersion objective). Data represent as mean ± SEM. ∗p < 0.05; ∗∗p < 0.01; ∗∗∗p < 0.001. (Mann-Whitney test).

In addition to NL63, indomethacin exerted a broad-spectrum antiviral activity against 229E, OC43, and SARS-CoV-2 in cell culture models, although the potency varies among different coronaviruses (Figure 1). For example, the EC50 value of 229E was 1.615 μM and SI was over 60 (Figure 1D), whereas the EC50 value of SARS-CoV-2 was 16.69 μM and SI was about 6 (Figure 1I). Primary human airway organoids (hAOs) represent an advanced model for studying virus infection and assessment of antiviral agents.^21^ Thus, we further validated the potency of indomethacin against pan-coronavirus in hAOs models. A potent inhibition of viral RNA replication was observed in pan-coronavirus-infected hAOs treated with indomethacin (Figures 2A, 2C, and 2E). Immunofluorescence staining of SARS-CoV-2 Npro or dsRNA confirmed the potent antiviral effect of indomethacin against pan-coronavirus (Figures 2B, 2D, and 2F). Overall, these results indicate that indomethacin possesses pan-coronavirus antiviral activity. To investigate which step(s) of the viral life cycle is blocked by indomethacin in pan-coronavirus infection, we also performed a time-of-drug-addition experiment (Figure S2A).^22^ Pre-treatment and treatment during virus inoculation had minor effects but inhibited coronavirus infection at the post-entry stage (Figures S2B–S2D).

### The anti-coronavirus effect of indomethacin is independent of the cyclooxygenase pathway

As an NSAID, indomethacin exerts anti-inflammatory effects through inhibition of cyclooxygenase-1 (COX-1) and cyclooxygenase-2 (COX-2) enzymes to prevent the production of prostaglandins (Figure S3A).^23^ Here we found that coronavirus infections can activate the expression of PTGS1 (encoding COX1) and PTGS2 (encoding COX2) at mRNA level (Figure S3B). To investigate whether the anti-coronavirus effect of indomethacin is via PTGS1/2, we used lentiviral shRNA vectors to stably knock down PTGS1 and PTGS2 gene in A549 cell line, respectively (Table S4). Two clones with optimal gene knockdown were selected for subsequent functional experimentation (Figure S3C). Compared with the control, knockdown of these two genes had no clear effect on coronavirus replication (Figure S3D), suggesting that PTGS1 and PTGS2 are not required for coronavirus replication. In addition, the anti-coronavirus activity of indomethacin was not affected in PTGS1- and PTGS2-knockdown cells (Figure S3E).

### Combining indomethacin with oral antiviral drug molnupiravir or nirmatrelvir results in synergistic antiviral activity

**Figure 3:**
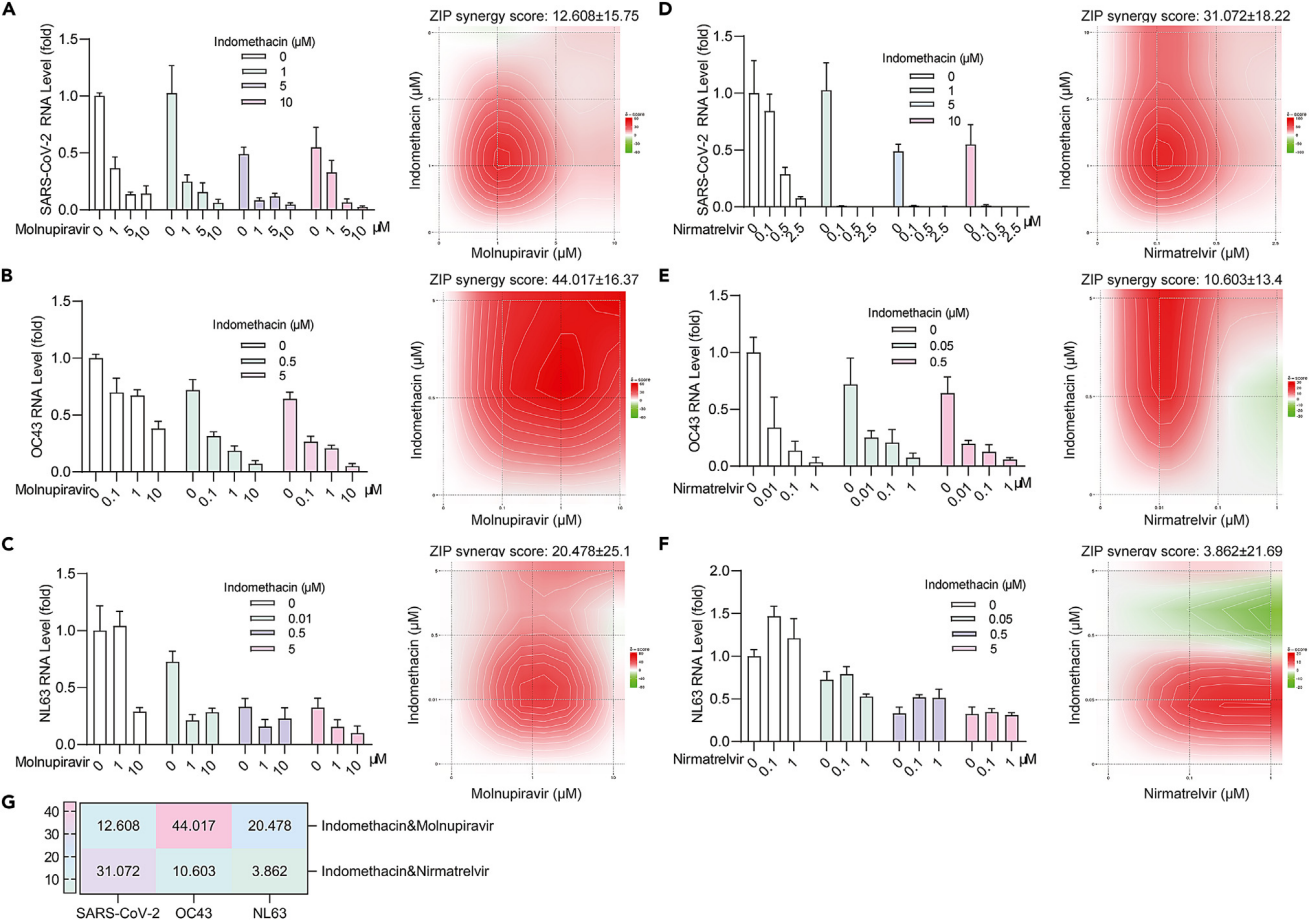
The combinational effects of indomethacin with molnupiravir or nirmatrelvir (A–C) The antiviral effects and synergy distribution of combining indomethacin with molnupiravir in NL63, OC43 or SARS-CoV-2 infected cell models (n = 4–8). (D–F) The antiviral effects and synergy distribution of combining indomethacin with nirmatrelvir in NL63, OC43 or SARS-CoV-2 infected cell models (n = 4–7). (G) The summary of ZIP synergy score of the combination of indomethacin with molnupiravir or nirmatrelvir in NL63, OC43 and SARS-CoV-2. Data are presented as mean ± SEM; ∗p < 0.05; ∗∗p < 0.01; ∗∗∗p < 0.001. (Mann-Whitney test).

The oral direct-acting antiviral drugs molnupiravir and nirmatrelvir have been widely used for treating COVID-19 patients as monotherapy.^24^ Since antiviral drug combination is a common strategy to enhance efficacy and prevent drug-resistance development, we evaluated the combinatory effects of molnupiravir and nirmatrelvir with indomethacin in cell culture models of SARS-CoV-2, OC43 and NL63 infections. In general, synergism was observed across different combinations (Figures 3A–3G), which is particularly strong when combining molnupiravir with indomethacin compared to indomethacin or molnupiravir alone (Figures 3A–3C). As an exception, nirmatrelvir has no antiviral activity against NL63, and thus the combination in this specific case is not meaningful in clinical study (Figure 3F).

### Drug screening identified that indomethacin potentiates interferon response

**Figure 4:**
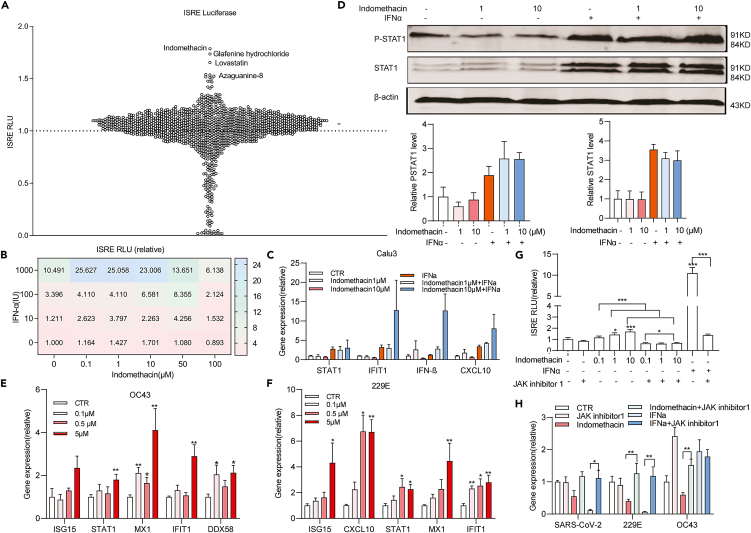
Drug screening identified that indomethacin potentiates interferon response by increasing STAT1 phosphorylation (A) Huh7-ISRE-Luc cells were treated with 1280 compounds at a concentration of 10 μM or DMSO vehicle control at the presence of 100 IU/mL IFN-α for 48 h. (B) Analysis of ISRE related firefly luciferase activity in Huh7-ISRE-Luc cells treated with the combination of different concentrations of indomethacin and IFN-α (1000IU/mL) for 48 h (n = 15–40). (C) Gene expression of ISGs in Calu-3 cells was quantified by qRT-PCR after treatment with the combination of indomethacin and IFN-α (1000IU/mL) for 48 h (n = 10–36). (D) Western blot analysis of total STAT1 or phosphorylated STAT1 (pSTAT1) expression in Calu-3 cells treated with the combination of indomethacin and IFN-α (1000IU/mL) for 48 h (n = 4–6). (E and F) Gene expression of ISGs in OC43 or 229E infected A549 cells were quantified by qRT-PCR after treatment with different concentrations of indomethacin for 48 h (n = 5). (G) Analysis of ISRE related firefly luciferase activity in Huh7-ISRE-Luc cells treated with indomethacin, IFN-α (1000 IU/mL) and/or JAK inhibitor 1 (10 μM) at different concentration for 48 h (n = 14–25). (H) qRT-PCR analysis of coronavirus RNA in cells treated with indomethacin, IFN-α (1000IU/mL) and/or JAK inhibitor 1 (10 μM) for 48 h (n = 4–9). Data are presented as mean ± SEM; ∗p < 0.05; ∗∗p < 0.01; ∗∗∗p < 0.001 (Mann-Whitney test) See also Figure S4.

IFN-α as a key member of type I interferons is a widely broad-spectrum antiviral agent and has also been clinically explored for treating COVID-19 patients.^25^^,^^26^ However, the efficacy of IFN-α monotherapy is often suboptimal but requires a combination to achieve improved efficacy. Aiming at identifying agents that augment the antiviral activity of IFN-α, we screened a library of 1,280 compounds (95% are FDA-approved drugs). We employed a transcriptional reporter system that can mimic interferon response with a luciferase gene that was driven by multiple interferon-stimulated response elements (ISRE-Luc). This model was treated with each compound at a concentration of 10 μM or DMSO vehicle control at the presence of 100 IU/mL IFN-α for 48 h. We found only a small subset of agents that exerted inhibitory or promoting effects on IFN-α-triggered luciferase activity (Figure 4A).

Coincidentally, we found indomethacin exerted the strongest augmenting effect, with over 1.7-fold increase of luciferase activity compared to IFN-α alone (Figure 4A). This effect was further confirmed using a series concentration of indomethacin and IFN-α (Figures 4B and S4). Activation of ISRE usually leads to the transcription of interferon-stimulated genes (ISGs) that contain this motif in their promoter regions. As expected, IFN-α stimulated the transcription of ISGs (e.g., IFIT1, CXCL10) in Calu-3 cells, whereas combination with indomethacin further activated their expression (Figure 4C). In the classical type I interferon-signaling pathway, Janus kinases phosphorylate STATs (in particular STAT1) to initiate the interferon response. Interestingly, we found indomethacin appears to further enhance IFN-α-triggered STAT1 phosphorylation but not the un-phosphorylated STAT1 (Figure 4D). These results may partially represent the underlying mechanism of how indomethacin potentiates antiviral interferon response.

Since indomethacin alone already moderately stimulated ISRE luciferase response (Figures 4B and S4), we further tested whether indomethacin can activate ISG transcription. In cells infected with OC43 or 229E, we found a moderate activation of the representative ISGs (Figures 4E and 4F). For example, 5 μM of indomethacin resulted in a 6-fold increase of CXCL10 gene expression (Figure 4F). To investigate whether this mediates the anti-coronavirus activity of indomethacin, we blocked the function of Janus kinase by JAK inhibitor 1. As expected, this completely blocked IFN-α-mediated ISRE activation and antiviral activity (Figures 4G and 4H). Surprisingly, it significantly reversed indomethacin-mediated ISRE activation and the anti-coronavirus activity of OC43 and 229E, although there was no significant difference in anti-SARS-CoV-2 activity (Figures 4G and 4H). These findings may prove that indomethacin inhibits pan-coronavirus activity by targeting JAK-STAT pathway.

### Combining indomethacin with IFN-α exerts synergistic antiviral effect except for OC43

**Figure 5:**
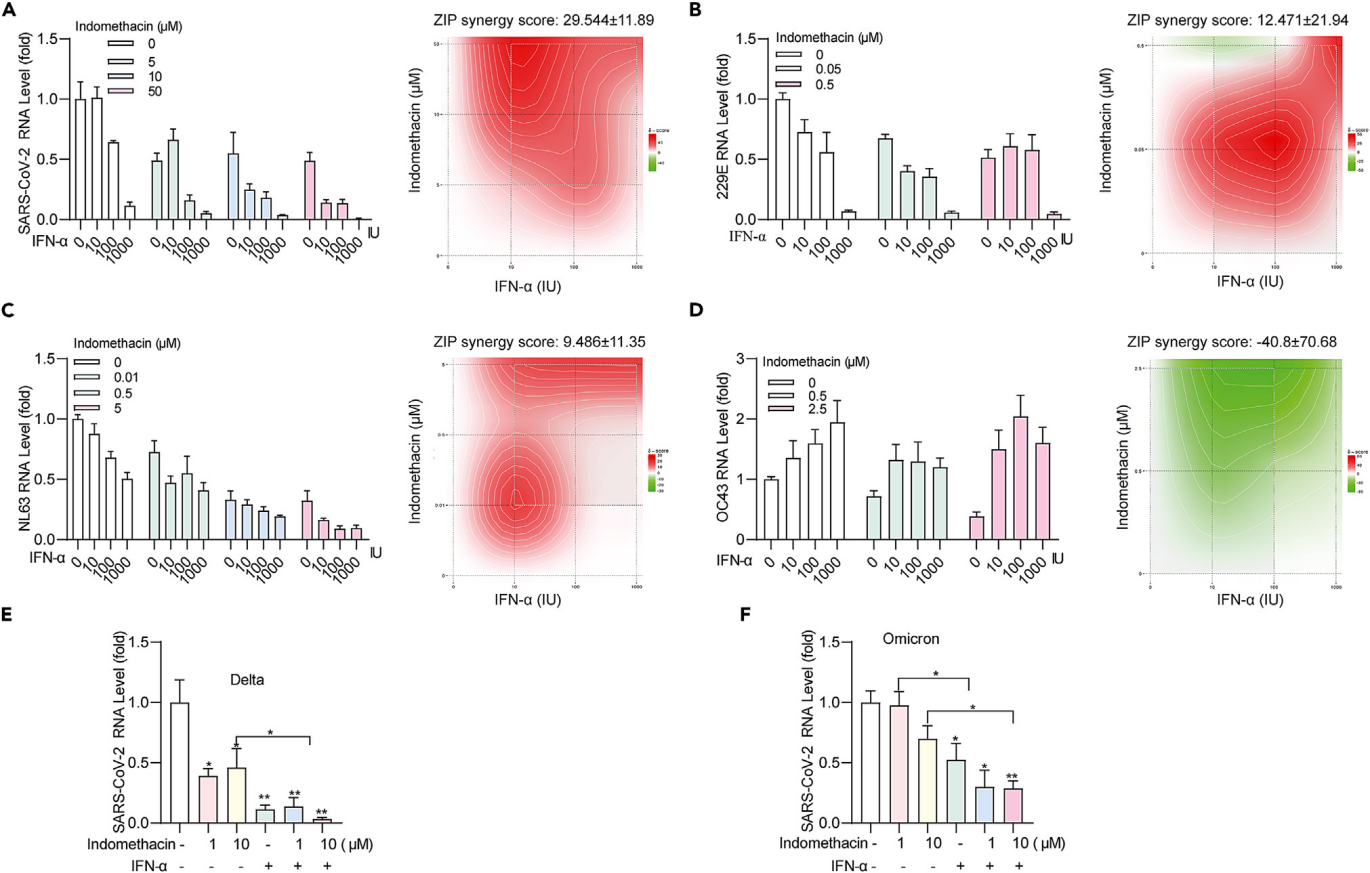
The combinational effects of indomethacin with IFN-α in cell models (A–D) The effects and synergy distribution of combining indomethacin with IFN-α in 229E, NL63, OC43 or SARS-CoV-2 infected cell models (n = 5–8). (E and F) The antiviral effects of combining indomethacin with IFN-α (1000 IU/mL) in SARS-CoV-2 Delta and Omicron variants infected cell models (n = 4–7). Data are presented as mean ± SEM; ∗p < 0.05; ∗∗p < 0.01; ∗∗∗p < 0.001. (Mann-Whitney test) See also Figure S5.

Next, we tested the combination of indomethacin with IFN-α on the replication of different coronaviruses in cell culture. We found that in NL63, 229E, and SARS-CoV-2 models, combining indomethacin with IFN-α exerted synergistic antiviral activity, especially on SARS-CoV-2 showing a ZIP synergy score around 30 (Figures 5A–5C). Furthermore, we also confirmed the enhanced antiviral effect of the combination in different variants (Delta and Omicron) of SARS-CoV-2 (Figures 5E and 5F). In contrast, this combination exhibited antagonistic effect in OC43 model (Figure 5D), which is in fact in accordance with the previous studies reporting the pro-viral effect of interferon on OC43 infection.^17^ Immunofluorescent staining of nucleocapsid protein (Npro) or viral dsRNA revealed similar results (Figure S5).

**Figure 6:**
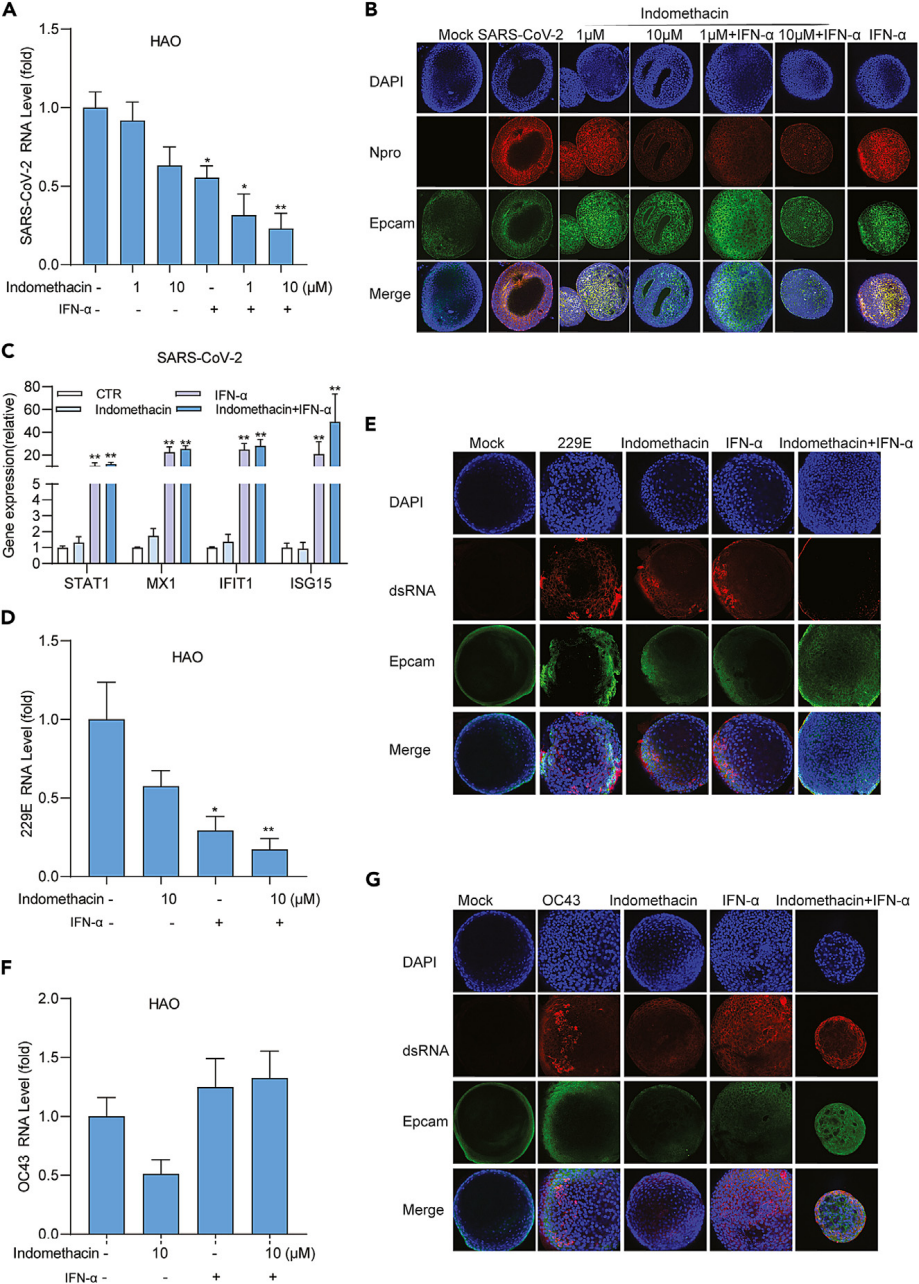
The combinational effects of indomethacin with IFN-α in hAOs models hAOs infected with SARS-CoV-2, 229E or OC43 respectively were treated with the combination of indomethacin and IFN-α (1000IU/mL) at indicated concentrations for 48 h. (A, D, and F) The effects on viral RNA was quantified by qRT-PCR (n = 5). (B, E, and G) Immunofluorescence analysis of Npro of SARS-CoV-2 or viral dsRNA in hAOs treated with the combination of indomethacin and IFN-α for 48 h. DAPI (blue) and Epcam (green) were applied to visualize nuclei and cytomembrane (Scale bar, 100 μm. 40x oil immersion objective). (C) Gene expression of ISGs in SARS-CoV-2 infected hAOs was quantified by qRT-PCR after treatment with the combination of indomethacin (10 μM) and IFN-α (1000 IU/mL) for 48 h (n = 5). Data represent as mean ± SEM. ∗p < 0.05; ∗∗p < 0.01; ∗∗∗p < 0.001. (Mann-Whitney test).

Interferon signaling provides an early innate defense against many viral infections. Secreted interferons bind to their receptors to initiate the Janus kinase signal transducer and activator of transcription (JAK-STAT) cascade to activate ISG transcription and establish an antiviral state.^12^^,^^27^ hAOs generated from tissue-resident stem cells and cultured in a 3D structure, which are not only much better at recapitulating the architecture, composition, diversity, organization, and functionality of cell types but also better suitable for studying virus-host interactions.^21^ Thus, we further explored the combination treatment of indomethacin with IFN-α in hAOs models. In SARS-CoV-2 infected hAOs, combining indomethacin with IFN-α significantly inhibited viral replication by both qRT-PCR quantification of viral RNA (Figure 6A) and immunofluorescence staining of Npro (Figure 6B). As expected, combining indomethacin with IFN-α also effectively activated the transcription of ISGs including STAT1, ISG15, IFIT1, and MX1 in hAOs (Figure 6C). SARS-CoV-2-triggered activation of interferon signaling functionally restricts viral replication but is not sufficient to completely defend the infection, whereas therapeutic combining indomethacin with IFN-α treatment harnesses this innate defense mechanism. This synergistic antiviral effect was further confirmed in 229E hAOs infection model (Figures 6D and 6E). Consistent with cell lines, OC43 hAO infection model also exerted antagonistic effect after treating with the combination of indomethacin with IFN-α (Figures 6F and 6G).

### Indomethacin exerted both anti-inflammatory and antiviral effects

**Figure 7:**
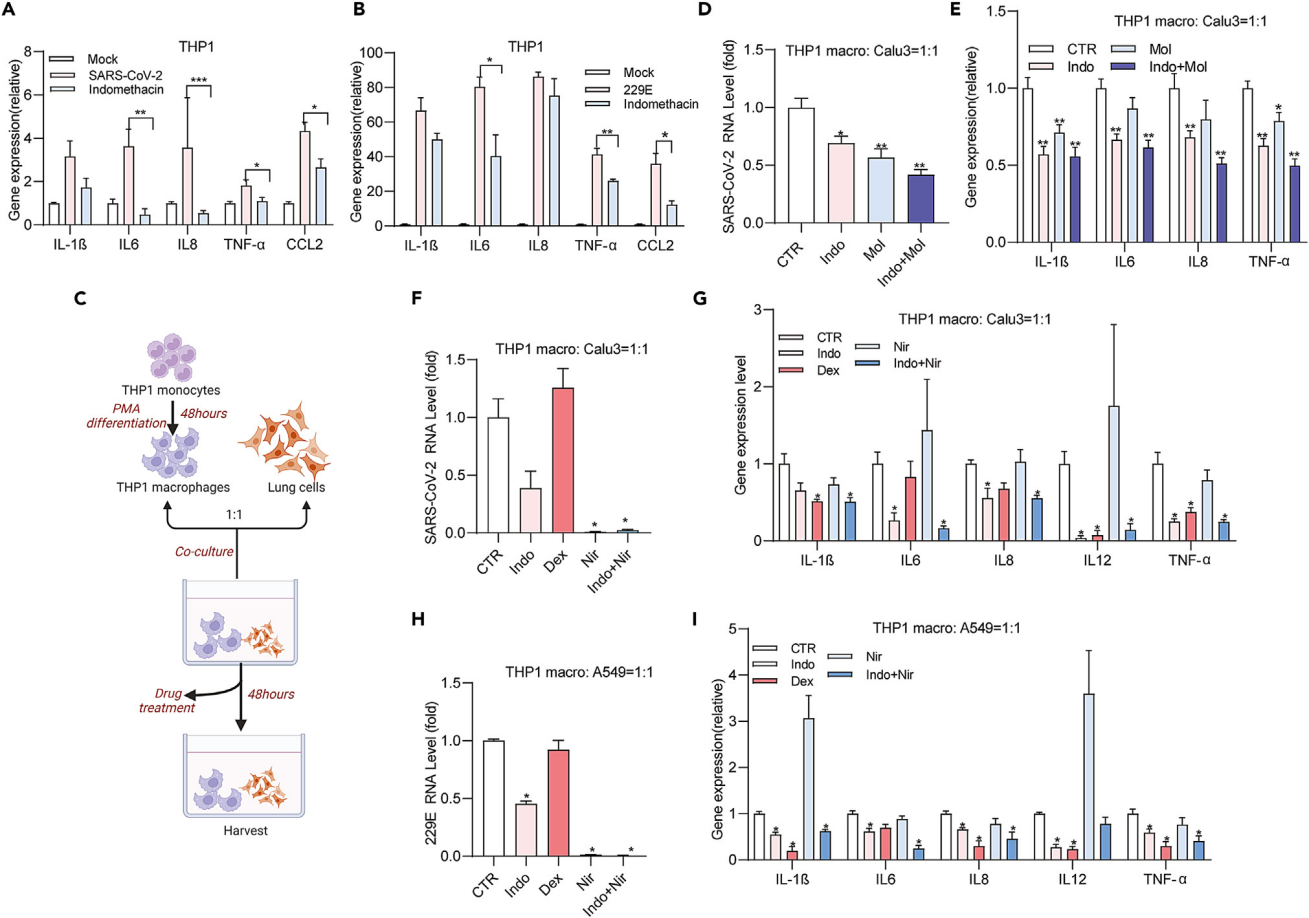
Indomethacin exerted both anti-inflammatory and antiviral effect against pan-coronavirus infection THP-1 macrophages inoculated with SARS-CoV-2 or 229E particles and treated with indomethacin (10 μM), dexamethasone(10 μM), nirmatrelvir (2.5 μM), or their combinations for 48 h. (A and B) mRNA levels of inflammation cytokines were quantified by qPCR (n = 5–15). (C) Schematic illustration of the co-culture system of human lung epithelial cells with THP-1 macrophages infected with coronaviruses. This illustration was prepared by using the web-based tool BioRender. The co-culture system was inoculated with SARS-CoV-2 particles and treated with indomethacin (10 μM), molnupiravir (10 μM) or their combination for 48 h. (D) mRNA levels of coronavirus were quantified by qPCR (n = 6). (E) mRNA levels of inflammatary cytokines were quantified by qPCR (n = 6). The co-culture system was inoculated with 229E or SARS-CoV-2 particles and treated with indomethacin (10 μM), dexamethasone (10 μM), nirmatrelvir (2.5 μM) or their combinations for 48 h. (F and H) mRNA levels of coronavirus were quantified by qPCR (n = 4–6). (G and I) mRNA levels of inflammatary cytokines were quantified by qPCR (n = 4). Data represent as mean ± SEM. ∗p < 0.05; ∗∗p < 0.01; ∗∗∗p < 0.001. (Mann-Whitney test) See also Figure S6 macro: macrophages, Indo: Indomethacin, Mol: Molnupiravr, Nir: Nirmatrelvir, Dex: Dexamethasone.

We next assessed the anti-inflammatory effect of indomethacin in macrophages. Interestingly, we found indomethacin exerted anti-inflammatory effect in coronavirus-infected macrophages (Figures 7A and 7B). Additionally, indomethacin also exerted anti-coronavirus effect in these macrophages (Figures S6A and S6B).

Given that macrophages are not the primary target of coronavirus infection, we then established a co-culture system of human lung cell lines with THP-1 macrophages to mimic host micro-environment for assessing combination therapy (Figure 7C). In this system, molnupiravir or nirmatrelvir treatment alone significantly inhibited coronavirus replication but not the expression of inflammatory genes (Figures 7D–7I). The anti-inflammation drug dexamethasone treatment alone significantly inhibited coronavirus-induced inflammatory gene expression but not coronavirus replication (Figures 7D–7I). Indomethacin alone simultaneously inhibited coronavirus-induced expression of inflammatory cytokines and coronavirus replication (Figures 7D–7I), without evidence of cross-interference. Among them, combination of indomethacin with molnupiravir or nirmatrelvir has the best inhibitory effect on virus infection and coronavirus-induced inflammatory gene expression (Figures 7D–7I). These results demonstrated a proof-of-concept of combining antiviral and anti-inflammatory drugs to simultaneously inhibit infection and inflammation.

## Discussion

FDA first approved the use of indomethacin in the 1960s to treat moderate to severe osteoarthritis, rheumatoid arthritis, and ankylosing spondylitis.^28^^,^^29^ Recently, indomethacin has been shown to have antiviral activity against a number of viruses, including herpesviruses, hepatitis B virus, vesicular stomatitis virus, and coronaviruses^30^^,^^31^^,^^32^^,^^33^ Previous studies have demonstrated indomethacin as an inhibitor of SARS-CoV, canine coronavirus, and SARS-CoV-2.^32^^,^^34^ This study further extended its antiviral activity against different variants of SARS-CoV-2 and three species of seasonal coronaviruses. In our SARS-CoV-2 infected human lung cell line, the EC50 value of indomethacin was determined as 16.369 μM by qRT-PCR quantification of viral genomes. This is more potent compared with previous studies reporting the EC50 value of over 90 μM.^35^^,^^36^ One possible explanation could be that these previous studies used the Vero cell line of monkey origin, whereas this study employed human cell lines. In addition to cell line models, we further confirmed the anti-coronavirus activity of indomethacin in hAOs.

Given the encouraging results obtained from experimental models, indomethacin has been tested in several clinical trials for treating COVID-19 patients.^37^ Meta-analysis of four studies showed a moderate improvement for recovery but did not reach statistical significance.^38^ Overall, indomethacin monotherapy is likely insufficient to effectively clear coronavirus infection in patients. Paxlovid (nirmatrelvir-ritonavir) and molnupiravir are two approved oral antiviral drugs for treating COVID-19. Although data from early clinical trials were very promising, real-world effectiveness of monotherapy with this antiviral treatment remains suboptimal.^39^ Furthermore, long-term use of antiviral monotherapy can induce viral mutagenesis and develop drug resistance, especially in immunocompromised patients.^40^^,^^41^ This may partially explain COVID-19 rebound in patients after completion of nirmatrelvir-ritonavir or molnupiravir treatment. In contrast, combinational antiviral treatment can exhibit synergism and prevent the development of drug-resistant strains.^9^ In this study, we found combination of indomethacin with molnupiravir or nirmatrelvir exerted synergistic antiviral activity in cell culture models with coronavirus infections. These results support further development of such combinations for treating coronavirus-infected patients, including validation in animal models and in clinical trials.

IFN-α is a broad-spectrum antiviral cytokine that has been widely used for treating a variety of viral diseases.^14^^,^^15^ Although IFN-α has been clinically explored for treating COVID-19 patients, it did not significantly improve the survival of hospitalized COVID-19 patients.^42^ Clinical experience of treating hepatitis C patients demonstrated that combination of IFN-α with ribavirin doubles the response rates, although ribavirin alone has minimal clinical efficacy.^43^ Mechanistically, IFN-α activates the JAK-STAT pathway to induce antiviral ISGs.^17^ Based on this mechanism, we employed a reporter assay aimed at identifying drugs that can further enhance interferon response and can be potentially used to combine with IFN-α. Coincidentally, through drug screening, we identified indomethacin as the most potent potentiator of interferon response, when combined with IFN-α. We further demonstrated that the combination of indomethacin with IFN-α exerted a synergistic antiviral effect on coronaviruses except for OC43. This is in fact in line with previous studies showing that OC43 can effectively antagonize antiviral interferon response and even explore it to facilitate entry into host cells.^17^ These results demonstrated the potential of combining indomethacin with IFN-α for treating coronavirus infection. However, further development of IFN-α-based combinational treatment should take into consideration of the specific coronavirus species. Previous studies employing computational docking and proteomic analysis found PGES2 (prostaglandin E synthase 2) rather than COX-2 interacted with viral NSP7 of epidemic coronaviruses.^44^^,^^45^ However, we found that inhibiting COX-1 and COX-2 enzymes—the classical anti-inflammatory targets of indomethacin had no effect on the anti-coronavirus activity of indomethacin. Consistently, previous studies have shown that other COX inhibitors ibuprofen and meloxicam also have no effect on SARS-CoV-2 replication.^46^ Interestingly, we found indomethacin alone is sufficient to moderately activate ISG transcription. Pharmacologically blocking the JAK-STAT pathway prevented indomethacin-triggered ISG transcription and the anti-coronavirus activity. These results suggest that activating cellular innate immunity by indomethacin (partially) contributes to its antiviral mechanism. However, it is likely that other mechanisms also contribute to the antiviral action of indomethacin, yet to be identified. Of note, blockade of COX-1 predisposes to gastric ulcers and bleeding, but inhibiting COX-2 reduces both pain and inflammation and blocks the vasodilatory and antiplatelet effects in the vascular wall.^23^ Recently, new indomethacin analogs which are more potent against COX-2 have been developed to overcome the non-selectivity and ulcer liability of indomethacin.^47^ It would be interesting to also test these new indomethacin analogs on coronavirus infection in the future.

As emphasized, patients with severe coronavirus infection are often accompanied with hyperinflammation primarily driven by macrophages, which is the key cause of morbidity and mortality.^48^ For these patients, we postulate that antiviral therapy alone is insufficient, but simultaneous inhibition of viral infection and pathological inflammation would be necessary to achieve improved outcome. Recent studies have reported that agents such as niclosamide and particular MEK inhibitors (targeting the MAPK pathway) can inhibit both SARS-CoV-2 replication and inflammatory response in experimental models.^49^^,^^50^ In a co-culture model of human lung cells with macrophages, we found that indomethacin has similar properties showing inhibition of inflammatory gene expression and coronavirus replication. However, we believe monotherapy of indomethacin would remain suboptimal. Thus, we tested the combination of indomethacin with molnupiravir or nirmatrelvir and demonstrated simultaneous inhibition of viral replication and inflammatory response in the co-culture system. Here, we demonstrated the advantages of this therapeutic approach for treating severe virus infections, but further validation in animal models is required before proceeding to clinical testing.

In summary, this study identified indomethacin as a pan-coronavirus inhibitor. It promotes the host antiviral response but inhibits inflammatory response. Combination of indomethacin with the oral antiviral drug molnupiravir or nirmatrelvir exerts synergetic antiviral activity and simultaneously inhibits inflammatory response. Because the experimental designs in this study consider the pan-coronavirus perspective, our findings are relevant not only to the currently circulating seasonal coronaviruses and SARS-CoV-2 but also to new variants of SARS-CoV-2 as well as new coronavirus species that may emerge in the future. Nevertheless, clinical implementations of these findings require further validation in animal models.

### Limitations of the study

Of note, there are some limitations in this study. Firstly, the pan-coronavirus antiviral activity of indomethacin was demonstrated in cell lines and human airway organoid models *in vitro*. Further validation in animal models is required before proceeding to clinical testing. Secondly, we found that activating cellular innate immunity contributes to the antiviral activity of indomethacin. However, other mechanisms may also contribute to the antiviral action of indomethacin, yet to be identified. Finally, we constructed a co-culture system of human epithelial cells with macrophages to test combination of antiviral and anti-inflammatory treatments. However, this model was based on immortalized cell lines. We believe this can be further improved for example by employing human airway organoids and primary macrophages.

## STAR★Methods

### Key resources table

**Table:** 

REAGENT or RESOURCE	SOURCE	IDENTIFIER
**Antibodies**		

Anti-double-stranded-RNA antibody (SCIONS J2 monoclonal antibody)	English&Scientific Consulting Kft	Cat# 10010200; RRID: AB_2651015
Anti-SARS-CoV-2-nucleocapsid protein	Thermo Fisher Scientific	Cat# MA5-29981; RRID: AB_2785780
Anti-EpCAM antibody	Abcam	Cat# ab71916; RRID: AB_1603782
Goat anti-Mouse IgG (H+L) Highly Cross-Adsorbed Secondary Antibody (Alexa Fluor Plus 594)	Thermo Fisher Scientific	Cat# A32742; RRID: AB_2762825
Anti-rabbit IgG (H+L), F(ab')2 Fragment (Alexa Fluor®488 Conjugate)	Bioké	Cat# 4412S; RRID: AB_1904025
Vectashield with DAPI	Thermo Fisher Scientific	Cat# 13285184; RRID: AB_2336790
Phospho-STAT1 (Tyr701)	Cell Signalling Technology	Cat# 7649; RRID: AB_10950970
STAT1	Cell Signalling Technology	Cat# 9172; RRID: AB_2198300
IRDye® 800CW Goat anti-Rabbit IgG Secondary Antibody	LI-COR	Cat# 926-32211; RRID: AB_621843
IRDye® 800CW Goat anti-Mouse IgG Secondary Antibody	LI-COR	Cat# 926-32210; RRID: AB_621842

**Bacterial and virus strains**		

NL63	Amsterdam Medical Center, Netherlands	GenBank: AY567487
OC43	ATCC	ATCC® VR-1558
229E	ATCC	ATCC® VR-740
SARS-CoV-2 Beta	Erasmus Medical Center, Netherlands	GenBank: MT270101
SARS-CoV-2 Omicron	Erasmus Medical Center, Netherlands	B.1.1.529
SARS-CoV-2 Delta	Erasmus Medical Center, Netherlands	B.1.617.2

**Chemicals, peptides, and recombinant proteins**		

Indomethacin	Sigma-Aldrich Chemie BV	I7378
Molnupiravir	MedChemExpress	HY-135853
Nirmatrelvir	MedChemExpress	HY-138687
Dexamethasone	Sigma-Aldrich Chemie BV	D4902
IFN-alpha 2b human	Merck	SRP4595
JAK inhibitor 1	Bio-Connect BV	sc-204021
Ibuprofen	MedChemExpress	HY-78131
Dimethyl Sulfoxide for Synthesis (DMSO)	Sigma-Aldrich Chemie BV	D2650
Puromycin	Sigma-Aldrich Chemie BV	P8833
MagNA Pure 96 External Lysis Buffer	Roche Diagnostics Nederland BV	6374913001
SB202190	Bio-Techne	1264/10
FGF10	Peprotech	AF-100-26-1000
Y27632	Bio-Techne	1254/10
FGF7	Tebu-Bio BV	100-19
Nicotinamide	Sigma-Aldrich Chemie BV	N0636
N-acetylcysteine	Sigma-Aldrich Chemie BV	A9165
B27	Thermo Fisher Scientific	12587001
DMEM high glucose w/Na pyruvate w/ Stable glutamine	VWR International BV	L0193-500
Advanced DMEM/F12	Life Technologies Europe BV	12634028
Opti-MEM I Reduced Serum Medium, no phenol red	Life Technologies Europe BV	11058021
HEPES (1M)	Life Technologies Europe BV	15630056
UltraGlutamine I (Alanyl-L-Glutamine) 200 mM (100X)	Westburg	LO BE17-605E/U1
Trypsin-EDTA (0.05%), phenol red	Life Technologies Europe BV	25300096
PenicillinStreptomycin Solution, liquid	Life Technologies Europe BV	11548876
Minimum Essential Medium (MEM) with Earle’s salts	Life Technologies Europe BV	32360034
Thiazolyl blue tetrazolium bromide (MTT]	Bio-Connect BV	CDX-T0186-G005
SYBR Select Master Mix for CFX-10 x	Thermo Fisher Scientific	4472954
Cultrex PathClear Reduced Growth Factor BME	Bio-Techne	3533-010-02
PMA(12-O-Tetradecanoylphorbol 13-acetate)	Sigma-Aldrich Chemie BV	P1585
RPMI 1640 (STABLE GLUTAMINE)	Westburg BV	L0498-500

**Critical commercial assays**		

NucleoSpin RNA (250 preps)	Bioke	MN 740955.250

**Experimental models: Cell lines**		

Human: sh55 A549 cell line	This paper	N/A
Human: sh59 A549 cell line	This paper	N/A
Human: sh60 A549 cell line	This paper	N/A
Human: sh63 A549 cell line	This paper	N/A

**Oligonucleotides**		

See Table S2 for primers sequences		

### Resource availability

#### Lead contact

Subsequent inquiries and requests for materials and chemicals should be sent to and will be fulfilled by the lead contact, Dr. Qiuwei Pan (q.pan@erasmusmc.nl).

#### Materials availability

A549 PTGS1/2 knock down cell models generated in this study, namely sh55, sh59,sh60 and sh63 A549 cells will be made available upon request.

### Experimental model and subject details

#### Reagents and antibodies

A library of 150 safe-in-man board-spectrum antiviral agents (https://drugvirus.info) were dissolved in DMSO or milli-Q water with a stock concentration of 10 mM.^51^^,^^52^ Molnupiravir and nirmatrelvir were dissolved in DMSO with the stock concentration of 100 mM. Human IFN-α was dissolved in PBS with the stock concentration of 1x10^7^ IU. JAK inhibitor 1 was dissolved in DMSO with a final concentration of 5 mg/mL.

#### Human airway organoids

Adult lung tissues were obtained from residual, tumor-free, material obtained at lung resection surgery for lung cancer. The Medical Ethical Committee of the Erasmus MC Rotterdam granted permission for this study (METC 2012-512). hAOs were cultured in airway organoid expansion medium (AEM) as undifferentiated status, based on advanced DMEM/F12 (Invitrogen), supplemented with 1% penicillin/streptomycin (Life Technologies), 1 M HEPES (Life Technologies), 200mM Ultraglutamine (Life Technologies), 2% (vol/vol) of B27 (Gibco), 1.25 mM N-acetylcysteine (Sigma-Aldrich), 10 mM Nicotinamide (Sigma-Aldrich), 10% (vol/vol) of R-spondin-1 (conditioned medium), 10% (vol/vol) of Noggin (conditioned medium), 100 ng/mL FGF10 (Peprotech), 25 ng/mL FGF7 (Peprotech), 1 μM SB202190 (Tocris), 500 nM A83-01 (Tocris) and 10 μM Y27632 (Sigma-Aldrich).

#### Cell lines

Monkey LLCMK-2 cells were cultured in minimal essential medium with Earle’s salt (MEM; Gibco, Grand Island, USA) containing 8% (vol/vol) heat-inactivated fetal calf serum (FCS, Sigma–Aldrich, St. Louis USA), 1% (vol/vol) nonessential amino acid (Sciencell, San Diego, California, USA), 0.1% (vol/vol) L-Glutamine (Lonza, Verviers, Belgium), 100 IU/mL Penicillin and 100 mg/mL Streptomycin (Gibco, Grand Island, USA). Multiple cell lines including human colon cancer cell line Caco-2, human hepatoma cell line Huh7, monkey kidney cell line Vero-E6 and adenocarcinomic human alveolar basal epithelial cell line A549 were cultured with Dulbecco's modifified Eagle medium (DMEM) (Lonza Biowhittaker, Verviers, Belgium) supplemented with 10% (vol/vol) heat-inactivated fetal calf serum (FCS, Sigma–Aldrich, St. Louis USA), 100 IU/mL Penicillin and 100 mg/mL Streptomycin (Gibco, Grand Island, USA). Human lung cancer cell line Calu-3 were cultured in advanced DMEM/F12 supplemented with 1% (vol/vol) GlutaMAXTM Supplement (Gibco, Grandisland, USA), 10 mM HEPES (Life Technologies), 100IU/mL Penicillin and 100 mg/mL Streptomycin (Gibco, Grand Island, USA) and 10% (vol/vol) heat-inactivated fetal calf serum (FCS, Sigma–Aldrich, St. Louis USA). The human monocytic cell lines THP-1 were cultured in RPMI 1640 Medium (Thermo Fisher) complemented with 10% (v/v) inactivated Fetal Bovine Serum with 100 IU/mL penicillin and 100 mg/mL streptomycin. For macrophage differentiation, THP-1 cells were treated with 20 ng/mL 12-myristate 13-acetate (PMA) at 37°C for 48 hours. Then cells were cultured for another 6 hours without PMA.

For the IFN-stimulated response element (ISRE) reporter model (Huh7-ISRE-Luc), human hepatic Huh7 cells were transduced with a lentiviral transcriptional reporter system expressing the firefly luciferase gene driven by a promoter containing multiple ISRE promoter elements (SBI Systems Biosciences, Mountain View, CA, USA). Luciferase activity represents ISRE promoter activation. All cells were maintained at 37°C in 5% CO_2_ incubator.

#### Viruses

NL63 stock was obtained from Amsterdam UMC location AMC, University of Amsterdam, The Netherlands. OC43 and 229E were bought from ATCC (USA). SARS-CoV-2 (isolate BetaCoV/Munich/BavPat1/2020; European Virus Archive Global #026V-03883, GenBank: MT270101; referred as a wild type strain), SARS-CoV-2 B.1.617.2 Delta variant (isolated from a patient) and SARS-CoV-2 B.1.1.529 Omicron variant (isolated from a patient) were used. Cell lines were analyzed by genotyping and confirmed to be mycoplasma negative.

#### Virus production assay

LLCMK-2 cells harboring the infectious NL63 were seeded into multi-well plates, culturing at 33°C, with 5% CO2 for 5-7 days. When over 50% of cells have cytopathic effect (CPE), NL63 particles were harvested by repeated freezing and thawing three times, filtered with 0.45 μm filters. Huh7 cells harboring the infectious OC43 or 229E were seeded into multi-well plates, culturing at 33°C, with 5% CO2 for 4-6 days. When over 50% of cells have cytopathic effect (CPE), OC43 or 229E particles were harvested by repeated freezing and thawing three times, then filtered with 0.45 μm filters. Calu-3 cells harboring the infectious SARS-CoV-2 were seeded into multi-well plates and incubating the cells at 37°C, with 5% CO2 for 3-4 days. The culture supernatant was cleared by centrifugation and stored in aliquots at −80°C. The virus titers were analyzed by TCID50 assay.

#### TCID50 assay

Viruses in the cultured cells and the supernatant were harvested through repeated freezing and thawing for three times. Virus titers were quantified by using a 50% tissue culture infectious dose (TCID50) assay. Briefly, ten-fold dilutions of NL63, 0C43, 229E and SARS-CoV-2 virus were inoculated onto LLCMK-2,Vero-E6, Huh7 or Calu-3 cells respectively, grown in a 96-well tissue culture plates at 2,000 cells/well. The plate was incubated at 33°C or 37°C for 4-7 days, and each well was examined under a light microscope for cytopathic effect (CPE). The TCID50 value was calculated by using the Reed-Muench method.

#### Drug treatment

Caco-2, LLCMK-2 and Huh7 cells were first inoculated with NL63 at an MOI of 0.1, and incubated at 33°C overnight, A549 cells or hAOs were first inoculated with 229E or OC43 at a MOI of 0.1, and incubated at 33°C 2 hours, Calu-3 cells or hAOs were first inoculated with SARS-CoV-2 at an MOI of 0.02, and incubated at 37°C 1 hour. The cells or hAOs were then washed twice to remove free virus particles and treated with several concentrations of different drugs for the indicated time period. THP-1 macrophages or co-culture system of THP-1 macrophages with lung cells were inoculated with 229E or SARS-CoV-2 particles at an MOI of 0.5 with or without combinations of different drugs for the indicated time period. Cells, total RNA or supernatant were collected for further analysis. When multiple drugs tested in the same experiment, the untreated group serves as the control for all these tested drugs.

#### MTT assay

Caco-2, LLCMK-2, Huh7, A549, Calu-3 cells were seeded into 96-well tissue culture plates (1×104cells/well), and then treated with the indicated compounds for 48 hours. Cells were incubated with 10 μL 5 mg/mL 3-(4,5-dimethyl-2-thiazolyl) -2,5-diphenyl-2H-tetrazolium bromide (MTT) for 3 hours, then replaced with 100 μL DMSO medium and incubated at 37°C for 30 minutes. The absorbance at 490 nm was recorded using a microplate absorbance reader (Bio-Rad, CA, USA).

#### RNA isolation, cDNA synthesis and qRT-PCR

Total RNA was isolated using Macherey-Nagel NucleoSpin® RNA II kit (Bioke, Leiden, The Netherlands) and quantified using a Nanodrop ND-1000 (Wilmington, DE, USA). cDNA was synthesized by using a cDNA synthesis kit (TaKaRa Bio, Inc., Shiga, Japan). Real-time PCR reactions were performed with SYBR-Green-based real-time PCR (Applied Biosystems®, Austin, USA) on a StepOnePlusTM System (Thermo Fisher Scientific LifeSciences). Glyceraldehyde 3-phosphate dehydrogenase (GAPDH) gene was used as a housekeeping gene. Relative gene expression of target gene was normalized to GAPDH using the formula 2-ΔΔCT, ΔΔCT = ΔCTsample - ΔCTcontrol (ΔCT = CT [target gene] - CT[GAPDH]). Template control and reverse transcriptase control were included in all qRT-PCR experiments, and all primers are listed in Table S1.

#### Gene knockdown by lentiviral vectors

Lentiviral pLKO knockdown vectors (Sigma-Aldrich) expressing shRNAs targeting PTGS1/2 and their appropriate controls were obtained from the Erasmus Biomics Center and were produced in HEK 293T cells. These shRNA sequences are listed in Table S3. Stable gene knockdown cells were generated after lentiviral vector transduction and selection in medium containing puromycin (3 μg/ml; Sigma). Lentiviral particles were harvested by repeated freezing and thawing 3 times and filtered by 0.45 μm filters. Naïve A549 cells were seeded into muti-well plates and culture medium was discarded when cell confluence was approximately 80%, followed by twice 1×PBS washing. Harvested viruses were added and incubated at 37°C with 5% CO2 for 48 hours. Then stable gene knockdown cells were selected and expanded by adding puromycin (3 μg/mL). The PTGS1/2 RNA level was quantified by using SYBR Green–based real-time PCR assay according to the manufacturer’s instructions. GAPDH was used as housekeeping gene to normalize gene expression using the 2-ΔΔCt method. The primer sequences were listed in Table S1.

#### Western blot assay

Proteins in cell lysates were heated at 95°C for 5-10 minutes, followed by loading onto a 10% sodium dodecyl sulfate polyacrylamide gel (SDS-PAGE), separated at 90 V for 120 minutes, and electrophoretically transferred onto a polyvinylidene difluoride (PVDF) membrane (pore size: 0.45 mm; Thermo Fisher Scientific Life Sciences) for 2 hours with an electric current of 250 mA. Subsequently, the membrane was blocked with blocking buffer (Li-Cor Biosciences) for 1 hour at room temperature. Membrane was followed by incubation with primary antibodies anti-PSTAT1, anti-STAT1 and anti-β-actin (1:1000) overnight at 4°C. The membrane was washed 3 times followed by incubation for 1 hour with anti-mouse or anti-rabbit IRDye-conjugated secondary antibodies (1:5000; Li-Cor Biosciences) at room temperature. After washing 3 times, protein bands were detected with Odyssey 3.0 Infrared Imaging System. 2.7.

#### Immunofluorescence staining

hAOs or Calu-3 cells cultured in the 8 μ-slide well chamber (cat. no. 80826; ibidi GmbH) were inoculated with SARS-CoV-2 at 0.02 MOI at 37°C 1 hour. hAOs or A549 cells cultured in 8 μ-slide well chamber were inoculated with OC43 or 229E at 0.1 MOI, respectively, and incubated at 33°C 3 hours. Caco-2 cells cultured in the 8 μ-slide well chamber (cat. no. 80826; ibidi GmbH) were inoculated with NL63 at 0.1 MOI at 37°C, overnight. The culture medium was then replaced by medium containing different concentrations of indomethacin, IFN-α or their combination and then they were cultured for another 48 hours. hAOs were washed in cold advanced DMEM/F12 medium 3 times to remove all basal matrix. Subsequently, hAOs were added into the CytoSpin II Cytocentrifuge (Shandon Scientifi Ltd, Runcorn, England) and spun down into slides at 1000 rpm for 5 minutes. Then hAOs or cells grown on 8 μ-slide well were fixed with 4% (w/v) paraformaldehyde (PFA) for 15 minutes at room temperature. The slides containing organoids or well plates with cells were then rinsed 3 times with PBS for 5 minutes each time, followed by permeabilizing with PBS containing 0.2% (vol/vol) tritonX100 for 15 minutes. Then the slides or plates were twice rinsed with PBS for 5 minutes, followed by incubation with blocking solution (5% donkey serum, 1% bovine serum albumin, 0.2% tritonX100 in PBS) at room temperature for 1 hour. Next, slides or plates were incubated in a humidity chamber with primary antibody diluted in blocking solution at 4°C overnight. Primary antibodies used in this study are as follows: Anti-SARS-CoV-2-nucleocapsid protein antibody (1:250, mouse mAb), anti-dsRNA antibody (1:200, mouse mAb), anti-EpCAM antibody (1:500, rabbit mAb). Excess primary antibodies were removed, and the slides or plates were washed 2 times for 10 minutes each in PBS containing 0.2% (vol/vol) tritonX100 and once for 10 minutes each in PBS prior to 1 hour incubation with 1:1000 dilutions of the anti-mouse IgG (H + L, Alexa Fluor® 594) and the anti-rabbit IgG (H + L, Alexa Fluor® 488). Nuclei were stained with DAPI (4, 6-diamidino-2-phenylindole; Invitrogen). Images were detected using Leica SP5 cell imaging system.

#### Co-culture of macrophages with lung cells harboring coronaviruses

THP-1 cells were treated with 20 ng/mL of PMA at 37°C for 48 hours. Then cells were cultured for another 6 hours without PMA. Co-culture of THP-1 macrophages with A549 or Calu-3 cells was established at a ratio of 1:1, mimicking the relative percentages of these cell populations in the human lung.^53^

#### Statistics

The statistical significance of differences between means was assessed with the Mann-Whitney test (GraphPad Prism; GraphPad Software Inc., La Jolla, CA). The threshold for statistical significance was defined as P < 0.05. Synergistic scores of drug combinations were analyzed by SynergyFinder 3.0.^54^

## Data Availability

• On request, the lead contact will share the original data reported in this article. • This article contains no original code. • Any extra data necessary to reanalyze the data given in this research is accessible upon request from the lead contact.

## References

[1] Zhou P., Yang X.L., Wang X.G., Hu B., Zhang L., Zhang W., Si H.R., Zhu Y., Li B., Huang C.L. (2020). A pneumonia outbreak associated with a new coronavirus of probable bat origin. Nature.

[2] Higgins V., Sohaei D., Diamandis E.P., Prassas I. (2021). COVID-19: from an acute to chronic disease? Potential long-term health consequences. Crit. Rev. Clin. Lab Sci..

[3] Petersen E., Koopmans M., Go U., Hamer D.H., Petrosillo N., Castelli F., Storgaard M., Al Khalili S., Simonsen L. (2020). Comparing SARS-CoV-2 with SARS-CoV and influenza pandemics. Lancet Infect. Dis..

[4] Telenti A., Hodcroft E.B., Robertson D.L. (2022). The Evolution and Biology of SARS-CoV-2 Variants. Cold Spring Harb. Perspect. Med..

[5] Li P., Liu J., Ma Z., Bramer W.M., Peppelenbosch M.P., Pan Q. (2020). Estimating Global Epidemiology of Low-Pathogenic Human Coronaviruses in Relation to the COVID-19 Context. J. Infect. Dis..

[6] Veiga A.B.G.d., Martins L.G., Riediger I., Mazetto A., Debur M.D.C., Gregianini T.S. (2021). More than just a common cold: Endemic coronaviruses OC43, HKU1, NL63, and 229E associated with severe acute respiratory infection and fatality cases among healthy adults. J. Med. Virol..

[7] Al-Tawfiq J.A., Sah R., Altawfiq K.J., Pan Q. (2023). Mpox-associated myopericarditis. New Microbes New Infect..

[8] Li J., Wang Y., Solanki K., Atre R., Lavrijsen M., Pan Q., Baig M.S., Li P. (2023). Nirmatrelvir exerts distinct antiviral potency against different human coronaviruses. Antivir. Res..

[9] Ianevski A., Yao R., Simonsen R.M., Myhre V., Ravlo E., Kaynova G.D., Zusinaite E., White J.M., Polyak S.J., Oksenych V. (2022). Mono- and combinational drug therapies for global viral pandemic preparedness. iScience.

[10] Rubin R. (2022). From Positive to Negative to Positive Again-The Mystery of Why COVID-19 Rebounds in Some Patients Who Take Paxlovid. JAMA.

[11] Wang L., Berger N.A., Davis P.B., Kaelber D.C., Volkow N.D., Xu R. (2022). COVID-19 Rebound after Paxlovid and Molnupiravir during January-June 2022. medRxiv.

[12] Wang W., Xu L., Su J., Peppelenbosch M.P., Pan Q. (2017). Transcriptional Regulation of Antiviral Interferon-Stimulated Genes. Trends Microbiol..

[13] Kamyshnyi A., Koval H., Kobevko O., Buchynskyi M., Oksenych V., Kainov D., Lyubomirskaya K., Kamyshna I., Potters G., Moshynets O. (2023). Therapeutic Effectiveness of Interferon-alpha2b against COVID-19 with Community-Acquired Pneumonia: The Ukrainian Experience. Int. J. Mol. Sci..

[14] Ianevski A., Yao R., Zusinaite E., Lello L.S., Wang S., Jo E., Yang J., Ravlo E., Wang W., Lysvand H. (2021). Synergistic Interferon-Alpha-Based Combinations for Treatment of SARS-CoV-2 and Other Viral Infections. Viruses.

[15] Ianevski A., Yao R., Lysvand H., Grodeland G., Legrand N., Oksenych V., Zusinaite E., Tenson T., Bjoras M., Kainov D.E. (2021). Nafamostat-Interferon-alpha Combination Suppresses SARS-CoV-2 Infection In Vitro and In Vivo by Cooperatively Targeting Host TMPRSS2. Viruses.

[16] Zielecki F., Weber M., Eickmann M., Spiegelberg L., Zaki A.M., Matrosovich M., Becker S., Weber F. (2013). Human cell tropism and innate immune system interactions of human respiratory coronavirus EMC compared to those of severe acute respiratory syndrome coronavirus. J. Virol..

[17] Zhao X., Guo F., Liu F., Cuconati A., Chang J., Block T.M., Guo J.T. (2014). Interferon induction of IFITM proteins promotes infection by human coronavirus OC43. Proc. Natl. Acad. Sci. USA.

[18] Liu F., Li L., Xu M., Wu J., Luo D., Zhu Y., Li B., Song X., Zhou X. (2020). Prognostic value of interleukin-6, C-reactive protein, and procalcitonin in patients with COVID-19. J. Clin. Virol..

[19] Hu B., Huang S., Yin L. (2021). The cytokine storm and COVID-19. J. Med. Virol..

[20] Hofmann H., Pyrc K., van der Hoek L., Geier M., Berkhout B., Pöhlmann S. (2005). Human coronavirus NL63 employs the severe acute respiratory syndrome coronavirus receptor for cellular entry. Proc. Natl. Acad. Sci. USA.

[21] Li P., Wang Y., Lamers M.M., Lavrijsen M., Iriondo C., de Vries A.C., Rottier R.J., Peppelenbosch M.P., Haagmans B.L., Pan Q. (2022). Recapitulating infection, thermal sensitivity and antiviral treatment of seasonal coronaviruses in human airway organoids. EBioMedicine.

[22] Daelemans D., Pauwels R., De Clercq E., Pannecouque C. (2011). A time-of-drug addition approach to target identification of antiviral compounds. Nat. Protoc..

[23] Cannon C.P., Cannon P.J. (2012). Physiology. COX-2 inhibitors and cardiovascular risk. Science.

[24] Li P., Wang Y., Lavrijsen M., Lamers M.M., de Vries A.C., Rottier R.J., Bruno M.J., Peppelenbosch M.P., Haagmans B.L., Pan Q. (2022). SARS-CoV-2 Omicron variant is highly sensitive to molnupiravir, nirmatrelvir, and the combination. Cell Res..

[25] Kalil A.C., Mehta A.K., Patterson T.F., Erdmann N., Gomez C.A., Jain M.K., Wolfe C.R., Ruiz-Palacios G.M., Kline S., Regalado Pineda J. (2021). Efficacy of interferon beta-1a plus remdesivir compared with remdesivir alone in hospitalised adults with COVID-19: a double-bind, randomised, placebo-controlled, phase 3 trial. Lancet Respir. Med..

[26] Ader F., DisCoVeRy Study Group (2022). An open-label randomized, controlled trial of the effect of lopinavir and ritonavir, lopinavir and ritonavir plus interferon-beta-1a, and hydroxychloroquine in hospitalized patients with COVID-19: final results. Clin. Microbiol. Infect..

[27] Xu L., Wang W., Peppelenbosch M.P., Pan Q. (2017). Noncanonical Antiviral Mechanisms of ISGs: Dispensability of Inducible Interferons. Trends Immunol..

[28] Hart F.D., Boardman P.L. (1963). Indomethacin: A New Non-Steroid Anti-Inflammatory Agent. Br. Med. J..

[29] Huskisson E.C., Taylor R.T., Burston D., Chuter P.J., Hart F.D. (1970). Evening indomethacin in the treatment of rheumatoid arthritis. Ann. Rheum. Dis..

[30] Ray N., Bisher M.E., Enquist L.W. (2004). Cyclooxygenase-1 and -2 are required for production of infectious pseudorabies virus. J. Virol..

[31] Gomeni R., Xu T., Gao X., Bressolle-Gomeni F. (2020). Model based approach for estimating the dosage regimen of indomethacin a potential antiviral treatment of patients infected with SARS CoV-2. J. Pharmacokinet. Pharmacodyn..

[32] Amici C., Di Caro A., Ciucci A., Chiappa L., Castilletti C., Martella V., Decaro N., Buonavoglia C., Capobianchi M.R., Santoro M.G. (2006). Indomethacin has a potent antiviral activity against SARS coronavirus. Antivir. Ther..

[33] Bahrami H., Daryani N.E., Haghpanah B., Moayyeri A., Moghadam K.F., Mirmomen S., Kamangar F. (2005). Effects of indomethacin on viral replication markers in asymptomatic carriers of hepatitis B: a randomized, placebo-controlled trial. Am. J. Gastroenterol..

[34] Chakraborty R., Bhattacharje G., Baral J., Manna B., Mullick J., Mathapati B.S., Abraham P., J M., Hasija Y., Ghosh A., Das A.K. (2022). In-silico screening and in-vitro assay show the antiviral effect of Indomethacin against SARS-CoV-2. Comput. Biol. Med..

[35] Kiani P., Scholey A., Dahl T.A., McMann L., Iversen J.M., Verster J.C. (2021). In Vitro Assessment of the Antiviral Activity of Ketotifen, Indomethacin and Naproxen, Alone and in Combination, against SARS-CoV-2. Viruses.

[36] Desantis J., Mercorelli B., Celegato M., Croci F., Bazzacco A., Baroni M., Siragusa L., Cruciani G., Loregian A., Goracci L. (2021). Indomethacin-based PROTACs as pan-coronavirus antiviral agents. Eur. J. Med. Chem..

[37] Ravichandran R., Mohan S.K., Sukumaran S.K., Kamaraj D., Daivasuga S.S., Ravi S.O.A.S., Vijayaraghavalu S., Kumar R.K. (2022). An open label randomized clinical trial of Indomethacin for mild and moderate hospitalised Covid-19 patients. Sci. Rep..

[38] van Kleef L.A., de Knegt R.J., Ayada I., Pan Q., Brouwer W.P. (2023). The Steatosis-associated fibrosis estimator (SAFE) score: validation in the general US population. Hepatol. Commun..

[39] Wong C.K.H., Au I.C.H., Lau K.T.K., Lau E.H.Y., Cowling B.J., Leung G.M. (2022). Real-world effectiveness of molnupiravir and nirmatrelvir plus ritonavir against mortality, hospitalisation, and in-hospital outcomes among community-dwelling, ambulatory patients with confirmed SARS-CoV-2 infection during the omicron wave in Hong Kong: an observational study. Lancet.

[40] Yip T.C.F., Lui G.C.Y., Lai M.S.M., Wong V.W.S., Tse Y.K., Ma B.H.M., Hui E., Leung M.K.W., Chan H.L.Y., Hui D.S.C., Wong G.L.H. (2023). Impact of the Use of Oral Antiviral Agents on the Risk of Hospitalization in Community Coronavirus Disease 2019 Patients (COVID-19). Clin. Infect. Dis..

[41] Li P., de Vries A.C., Kamar N., Peppelenbosch M.P., Pan Q. (2022). Monitoring and managing SARS-CoV-2 evolution in immunocompromised populations. Lancet Microbe.

[42] Buchynskyi M., Kamyshna I., Lyubomirskaya K., Moshynets O., Kobyliak N., Oksenych V., Kamyshnyi A. (2023). Efficacy of interferon alpha for the treatment of hospitalized patients with COVID-19: A meta-analysis. Front. Immunol..

[43] Pan Q., Tilanus H.W., Janssen H.L.A., van der Laan L.J.W. (2011). Ribavirin enhances interferon-stimulated gene transcription by activation of the interferon-stimulated response element. Hepatology.

[44] Terracciano R., Preianò M., Fregola A., Pelaia C., Montalcini T., Savino R. (2021). Mapping the SARS-CoV-2-Host Protein-Protein Interactome by Affinity Purification Mass Spectrometry and Proximity-Dependent Biotin Labeling: A Rational and Straightforward Route to Discover Host-Directed Anti-SARS-CoV-2 Therapeutics. Int. J. Mol. Sci..

[45] Gordon D.E., Hiatt J., Bouhaddou M., Rezelj V.V., Ulferts S., Braberg H., Jureka A.S., Obernier K., Guo J.Z., Batra J. (2020). Comparative host-coronavirus protein interaction networks reveal pan-viral disease mechanisms. Science.

[46] Chen J.S., Alfajaro M.M., Chow R.D., Wei J., Filler R.B., Eisenbarth S.C., Wilen C.B. (2021). Non-steroidal anti-inflammatory drugs dampen the cytokine and antibody response to SARS-CoV-2 infection. J. Virol..

[47] Abdellatif K.R.A., Abdelall E.K.A., Elshemy H.A.H., El-Nahass E.S., Abdel-Fattah M.M., Abdelgawad Y.Y.M. (2021). New indomethacin analogs as selective COX-2 inhibitors: Synthesis, COX-1/2 inhibitory activity, anti-inflammatory, ulcerogenicity, histopathological, and docking studies. Arch. Pharm..

[48] Fajgenbaum D.C., June C.H. (2020). Cytokine Storm. N. Engl. J. Med..

[49] de Almeida L., da Silva A.L.N., Rodrigues T.S., Oliveira S., Ishimoto A.Y., Seribelli A.A., Becerra A., Andrade W.A., Ataide M.A., Caetano C.C.S. (2022). Identification of immunomodulatory drugs that inhibit multiple inflammasomes and impair SARS-CoV-2 infection. Sci. Adv..

[50] Faist A., Schloer S., Mecate-Zambrano A., Janowski J., Schreiber A., Boergeling Y., Conrad B.C.G., Kumar S., Toebben L., Schughart K. (2023). Inhibition of p38 signaling curtails the SARS-CoV-2 induced inflammatory response but retains the IFN-dependent antiviral defense of the lung epithelial barrier. Antivir. Res..

[51] Andersen P.I., Ianevski A., Lysvand H., Vitkauskiene A., Oksenych V., Bjørås M., Telling K., Lutsar I., Dumpis U., Irie Y. (2020). Discovery and development of safe-in-man broad-spectrum antiviral agents. Int. J. Infect. Dis..

[52] Ianevski A., Zusinaite E., Kuivanen S., Strand M., Lysvand H., Teppor M., Kakkola L., Paavilainen H., Laajala M., Kallio-Kokko H. (2018). Novel activities of safe-in-human broad-spectrum antiviral agents. Antivir. Res..

[53] Travaglini K.J., Nabhan A.N., Penland L., Sinha R., Gillich A., Sit R.V., Chang S., Conley S.D., Mori Y., Seita J. (2020). A molecular cell atlas of the human lung from single-cell RNA sequencing. Nature.

[54] Ianevski A., Giri A.K., Aittokallio T. (2022). SynergyFinder 3.0: an interactive analysis and consensus interpretation of multi-drug synergies across multiple samples. Nucleic Acids Res..

