# Supplementary material for: Combating pan-coronavirus infection by indomethacin through simultaneously inhibiting viral replication and inflammatory response
Source: iScience. 2023 Aug 16;26(9):107631. doi: 10.1016/j.isci.2023.107631 (PMC10474465; doi:10.1016/j.isci.2023.107631)
Supplement: Document S1. Figures S1–S6 and Tables S1–S4 [file mmc1.pdf]

iScience, Volume *26*

Supplemental information

Combating pan-coronavirus infection

by indomethacin through simultaneously inhibiting

viral replication and inflammatory response

Yining Wang, Pengfei Li, Lei Xu, Annemarie C. de Vries, Robbert J. Rottier, Wenshi Wang, Marie-Rose B.S. Crombag, Maikel P. Peppelenbosch, Denis E. Kainov, and Qiuwei Pan

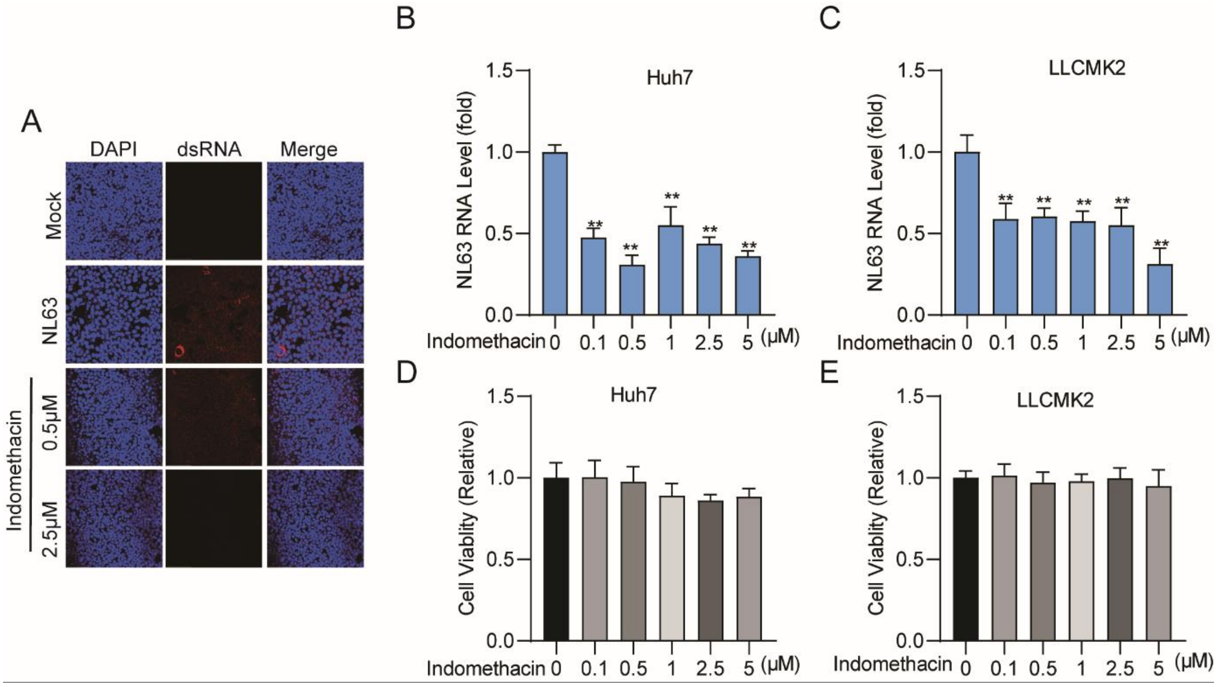

Figure S1. **The effects of indomethacin treatment on different cells inoculated with NL63 infectious particles, related to Figure 1.** (A) Immunofluorescence staining of dsRNA in NL63 infected Caco-2 cells treated with or without different concentrations of indomethacin. DAPI (blue) was applied to visualize nuclei. (Scale bar, 100 μm. 40x oil immersion objective). (B) and (C) Huh7 or LLCMK-2 cells infected with NL63 treated with different concentrations of indomethacin for 48 hours. The effects were quantified by qRT-PCR on viral RNA (n=6-7) (D) and (E) Huh7 or LLCMK-2 cells treated with different concentrations of indomethacin for 48 hours. Cytotoxicity was determined by MTT assay (n=15-16). Data represent as mean ± SEM. *P < 0.05; **P < 0.01; ***P < 0.001. (Mann-Whitney test)

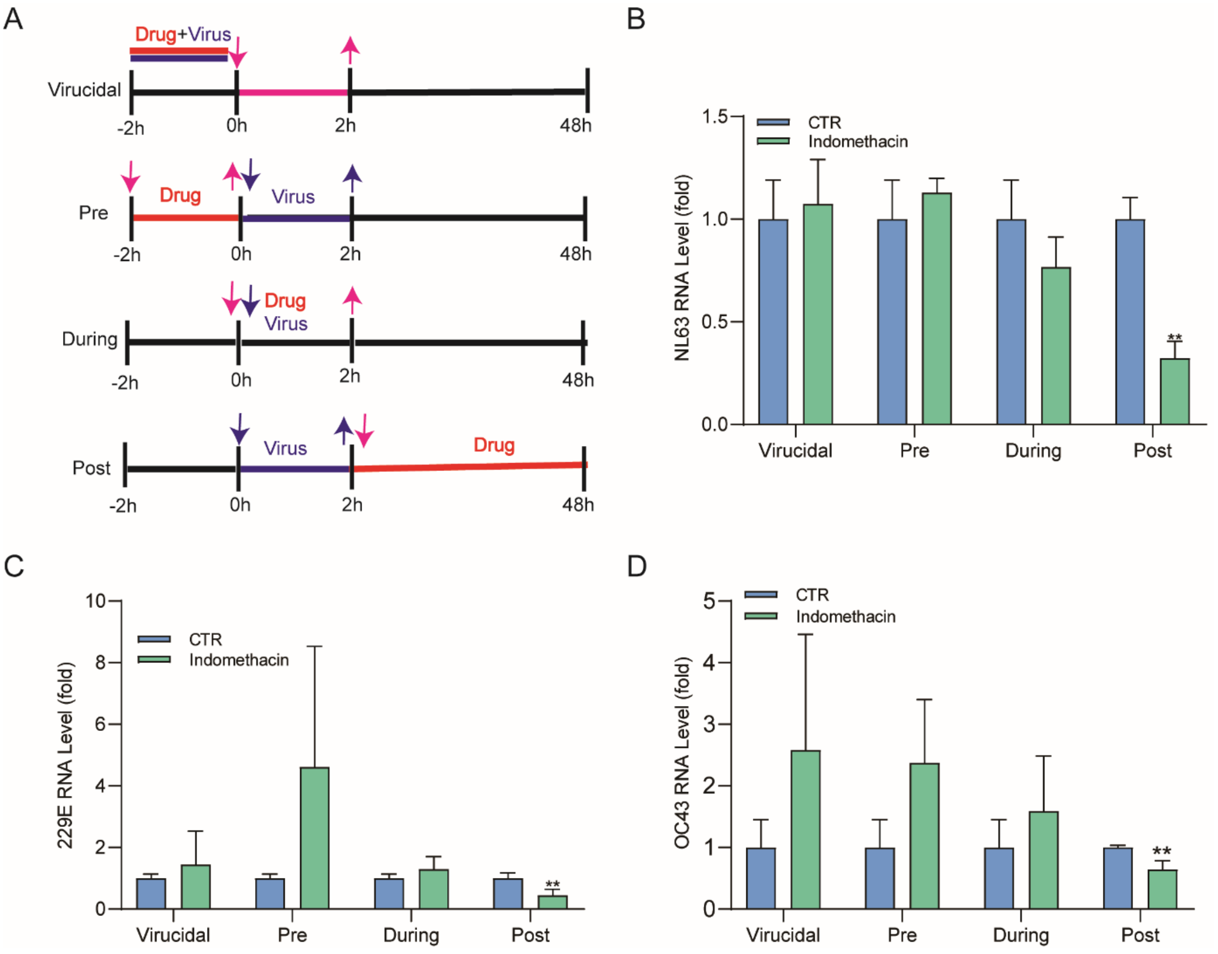

Figure S2. Time-of-addition analysis of the antiviral activity of indomethacin, related to Figure 1. (A) Schematic illustration of the time-of-addition experiment. (B), (C) and (D) Caco-2 or A549 cells were infected with NL63, 229E or OC43 at an MOI of 0.5 for 2 hours (0–2 hours) respectively. 5 μM indomethacin was introduced at different time points, designated as virucidal, pretreatment (pre), during treatment (during) or post-treatment (post). The inhibitory effect of indomethacin in each group was determined by qRT-PCR. Data represent as mean ± SEM. *$P < 0.05$; **$P < 0.01$; ***$P < 0.001$. (Mann-Whitney test)

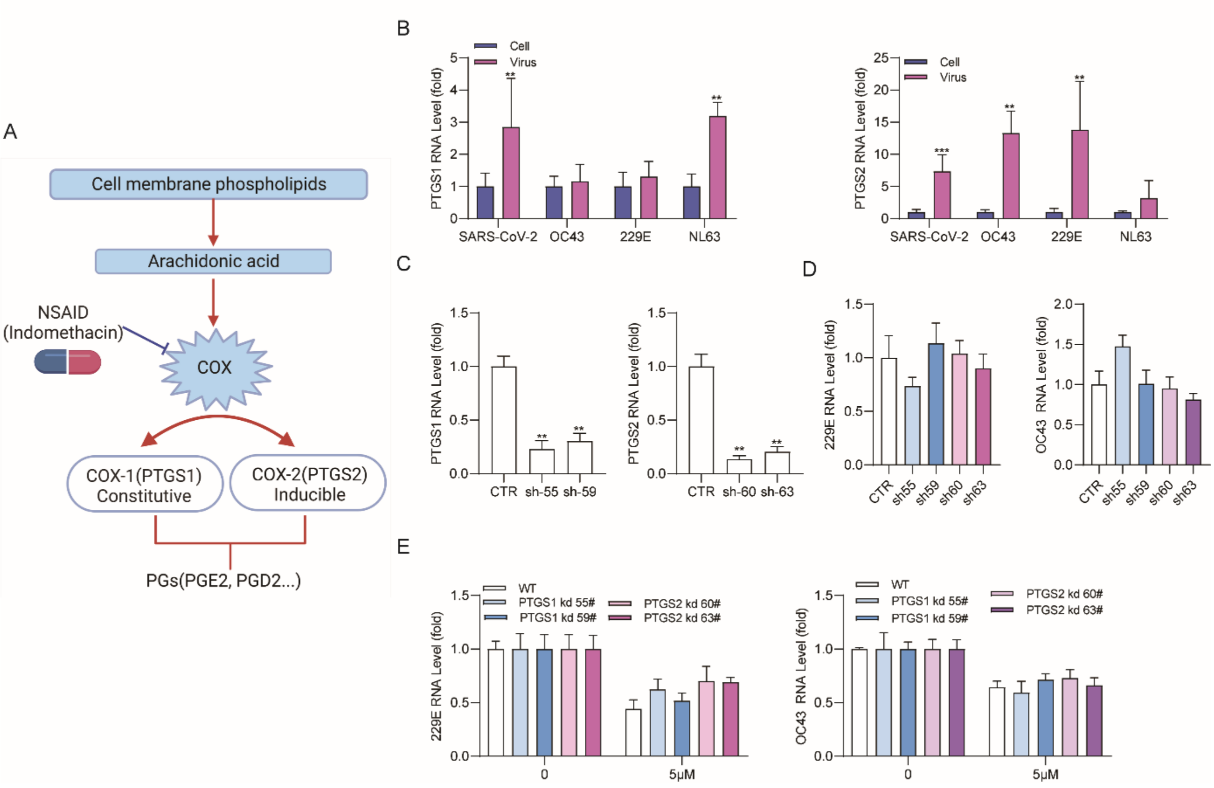

Figure S3. The anti-coronavirus effects of indomethacin is independent of the COX signaling pathway, related to Figure 1. (A) Schematic overview of the COX pathway targets of indomethacin. The figure was prepared by using the web-based tool BioRender. (B) Caco-2, A549 or Calu-3 cells were treated with or without NL63, 229E, OC43 or SARS-CoV-2 respectively. The expression level of PTGS1 and PTGS2 in pan-coronavirus infected cells were quantified by qPCR (n = 5-8). (C) Knockdown of PTGS1/2 by lentiviral shRNA vectors. The effects of 5 shPTGS1 or 5 shPTGS2 clones on PTGS1/2 expression were quantified by qRT-PCR. The expression of control vector transduced cells was set as 1 (n=5-6). (D) The effects of selected PTGS1/2 knockdown clones on cellular 229E or OC43 RNA levels in 229E or OC43 infected A549 cell models. Viral RNA was quantified by qRT-PCR (n = 6). (E) PTGS1/2 knockdown and control A549 cells infected with OC43 or 229E were treated with or without 5 μM indomethacin for 48 hours. The effect on viral RNA was quantified by qRT-PCR (n=6). Data represent as mean ± SEM. $^*P < 0.05$; $^{**}P < 0.01$; $^{***}P < 0.001$. (Mann-Whitney test)

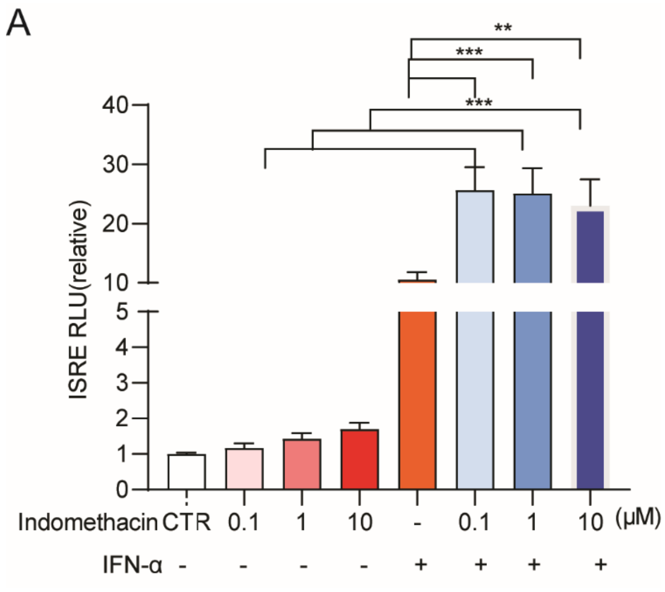

Figure S4. The combination of indomethacin with IFN-α stimulated ISRE related luciferase activity, related to Figure 4. Huh7-ISRE-Luc cells were treated with the combination of different concentrations of indomethacin with IFN-α (1000IU/mL) for 48hours (n=15-40). Data represent as mean ± SEM. *P < 0.05; **P < 0.01; ***P < 0.001. (Mann-Whitney test)

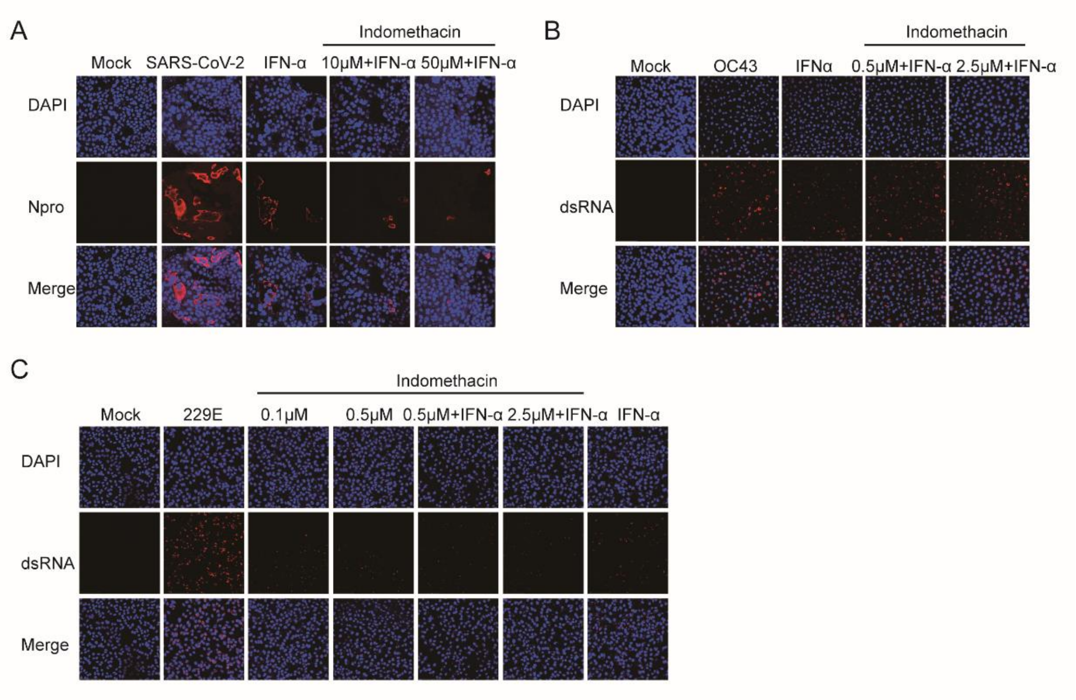

Figure S5. The effects of the combination treatment of indomethacin with IFN-α on cells inoculated with coronavirus infectious particles, related to Figure 5. (A), (B) and (C) Calu-3 or A549 cells infected with SARS-CoV-2, 229E or OC43 respectively and treated with the combination of indomethacin with IFN-α at indicated concentrations for 48 hours. Immunofluorescence analysis of Npro of SARS-CoV-2 or dsRNA. DAPI (blue) was applied to visualize nuclei. (Scale bar, 100 μm. 40x oil immersion objective).

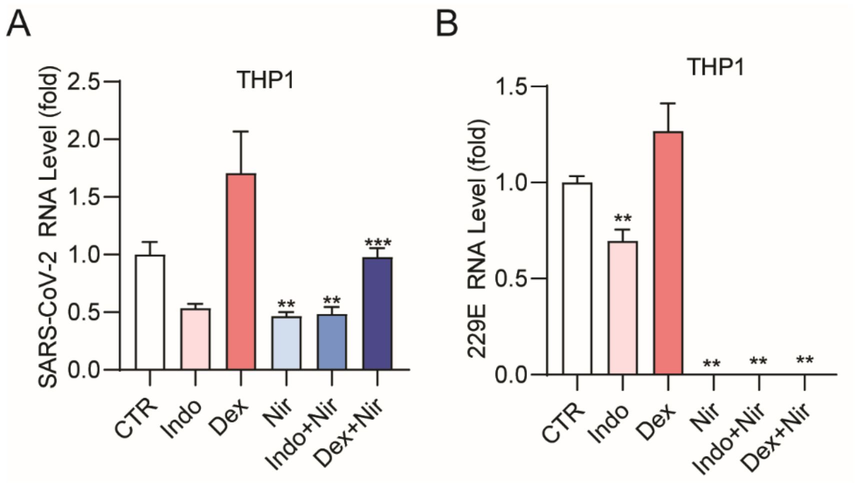

Figure S6. Indomethacin exerted antiviral effect against pan-coronavirus infection, related to Figure 7. (A) and (B) THP-1 macrophages inoculated with SARS-CoV-2 or 229E particles and treated with indomethacin (10μM), dexamethasone(10μM), nirmatrelvir (2.5μM) or their combination for 48 hours. mRNA levels of SARS-CoV-2 or 229E were quantified by qPCR (n=5-8). Data represent as mean ± SEM. *P < 0.05; **P < 0.01; ***P < 0.001. (Mann-Whitney test) Indo: Indomethacin, Mol: Molnupiravr, Nir: Nirmatrelvir, Dex: Dexamethasone.

Table S1 List of the library of broad-spectrum antiviral agents, related to Figure 1

1 Abamectin	51 Indomethacin	101 Diltiazem
2 ABT-263	52 Itraconazole	102 Teriflunomide
3 Acetylsalicylic acid	53 Ivermectin	103 Clomiprade hydrochloride
4 Aciclovir	54 Kasugamycin	104Chlorpromazine hydrochloride
5 Amiloride	55 Lamivudine	105 Quinacrine dihydrochloride
6 Amiodarone	56 Leflunomide	106 Toremifene
7 Amodiaquine	57 Lanatoside C (Isolanid)	107 Ibersartan
8 Apilimod	58 Lobucavir	108 Ranitidine
9 Arbidol	59 Lopinavir	109 Stanozolol
10 Artesunate	60 Lovastatin	110 Bumetanide
11 Azacitidine	61 Luteolin	111 Dalbavancin
12 Azithromycin	62 Manidipine	112 Remdesivir
13 BDA-366	63 Maribavir	113 Trametinib
14 Bepridil	64 Memantine	114 Acetylcysteine
15 Berberine	65 Metformin	115 Verdinexor
16 Bortezomib	66 Minocycline	116 Nicotine
17 Brequinar	67 Mitoxantrone	117 Mefloquine
18 Bromocriptine	68 Mycophenolic acid	118 Monensin
19 Caffeine	69 Nafamostat	119 Bemcentinib
20 Camostat	70 Nelfinavir	120 Daclasavir
21 Camptothecin	71 Nitazoxanide	121 Ozanimod
22 Chloroquine	72 Niclosamide	122 Osimertinib
23 Cidofovir	73 Novobiocin	123 Simeprevir
24 Clofarabine	74 Obatoclax	124 Pralatrexate
25 Cyclosporine	75 Omeprazole	125 Cycloheximide
26 Dasatinib	76 Pentosan polysulfate	126 Halofantrine
27 Dibucaine	77 Pirlindole	127 Mandipine
28 Diphyllin	78 Quinine	128 Baricitinib
29 Doxycycline	79 Raloxifene	129 AG-1478
30 DFMO	80 Rapamycin	130 Batimastat

31 Emetine	81 Ribavirin	131 Cepharanthine
32 Emodine	82 Ritonavir	132 EIDD-2801
33 Erlotinib	83 Regorafenib	133 Ilomastat
34 Esomeprazole	84 Simvastatin	134 Marimastat
35 Ezetimibe	85 Sofosbuvir (PSI-7977)	135 Vemurafenib
36 Famciclovir	86 Suramin	136 n6-(delta 2-isopentenyl)-adenine
37 Favipiravir	87 Tamoxifen	137 Ciclesonide
38 Fenretinide (4-HPR)	88 Teicoplanin	138 Vidarabine
39 Flavopiridol	89 Tenofovir	139 Digoxin
40 Fluoxetine	90 Topotecan	140 Dexamethasone
41 Fluvastatin	91 Trifluridine	141 Merimepodib
42 Formoterol	92 Valacyclovir	142 Pacritinib
43 Foscarnet	93 Verapamil	143 Digitoxin
44 Ganciclovir	94 Vidarabine	144 Oubain
45 Gefitinib	95 Bro mhexine HCl	145 Benidipine
46 Gemcitabine	96 Bromcriptine mesilate	146 Saquinavir
47 Glycyrrhizin	97 Cefoperazone acid	147 Atovaquone
48 Homoharringtonine	98 Moxalactam sodium salt	148 Lercanidipine
49 Hydroxychloroquine	99 Anisomycin	149 Octocrylene
50 Imatinib	100 Benzatropine	150 Permethrin

Table S2   Primers sequences, related to Figure1-7

Gene name	F-sequence (5' to 3')	R-sequence (5' to 3')
Human GAPDH	GTCTCCTCTGACTTCAACAGCG	ACCACCCTGTTGCTGTAGTAGCCA A
NL63	CTTCTGGTGACGCTAGTACAGCTTAT	AGACGTCGTTGTAGATCCCTAACAT
229E	GTCGTCAGGGTAGAATACCTTA	CCCGTTTGCGCTTTCTAGT
OC43	AGCAACCAGGCTGATGTCAATACC	AGCAGACCTTCCTGAGCCTTCAAT
SARS-CoV-2	CAATGGTTTAACAGGCACAGG	CTCAAGTGTCTGTGGATCACG
PTGS1	GATGAGCAGCTTTTCCAGACGAC	AACTGGACACCGAACAGCAGCT
PTGS2	CGGTGAAACTCTGGCTAGACAG	GCAAACCGTAGATGCTCAGGGA
IL-1β	CCACAGACCTTCCAGGAGAATG	GTGCAGTTCAGTGATCGTACAGG
TNF-α	CTCTTCTGCCTGCTGCACTTTG	ATGGGCTACAGGCTTGTCACTC
IL-6	AGACAGCCACTCACCTCTTCAG	TTCTGCCAGTGCCTCTTTGCTG
IL-8	GAGAGTGATTGAGAGTGGACCAC	GAGAGTGATTGAGAGTGGACCAC
IL-12	GACATTCTGCGTTCAGGTCCAG	CATTTTTGCGGCAGATGACCGTG
CCL2	AGAATCACCAGCAGCAAGTGTCC	TCCTGAACCCACTTCTGCTTGG
STAT1	ATGGCAGTCTGGCGGCTGAATT	CCAAACCAGGCTGGCACAATTG
IFIT1	GCCTTGCTGAAGTGTGGAGGAA	ATCCAGGCGATAGGCAGAGATC

IFN β	CTTGGATTCCTACAAAGAAGCAGC	TCCTCCTTCTGGAACTGCTGCA
CXCL10	GGTGAGAAGAGATGTCTGAATCC	GTCCATCCTTGGAAGCACTGCA
MX1	GGCTGTTTACCAGACTCCGACA	CACAAAGCCTGGCAGCTCTCTA
ISG15	CTCTGAGCATCCTGGTGAGGAA	AAGGTCAGCCAGAACAGGTCGT

Table S3 Results of 22 drug candidates, related to Figure 1

Agent	NL63 RNA (Relative)	MTT (Relative)
Berberine	0.2202645	0.920275443
Fenretinide (4-HPR)	0.232965216	0.73645077
Fluvastatin	0.21377877	1.006255895
Formoterol	0.277667757	0.912570324
Foscarnet	0.276288977	0.97880193
Ganciclovir	0.289208654	0.974139253
Gemcitabine	0.162325213	0.748098482
Indomethacin	0.30630565	0.8848116
Lamivudine	0.207906497	0.898762316
Luteolin	0.145762897	0.9198254
Memantine	0.132900735	0.963517689
Ritonavir	0.218609725	1.020802036
Sofosbuvir (PSI-7977)	0.168530808	1.084729269
Tamoxifen	0.216235768	0.817966596
Marimastat	0.05667232	0.917608005
Teriflunomide	0.255078328	1.215824291
Cycloheximide	0.09432894	1.064418116
Ivermectin	0.274562531	0.801332214
Ciclesonide	0.144053272	0.894157947
Digitoxin	0.29262237	0.594111243
Lercanidipine	0.12977902	1.008770815
Remdesivir	0.227423019	0.93061087

From the screening, 22 drugs were identified with more than 70% inhibition on NL63 replication (The relative NL63 RNA level is equal or less than 0.3) and less than 50% inhibition on cells (The relative MTT value is equal or more than 0.5).

Table S4 shRNA sequences of PTGS1 and PTGS2, related to Figure 1

ID	Symbol	TargetTaxonId	TargetSeq	OligoSeq
12155	PTGS1	Human	CGTGAGCTATTACACTCGTAT	CCGGCGTGAGCTATTACACTCGTATCTCGAGATACGAGTGTAATAGCTCACGTTTTTG
12156	PTGS1	Human	CGCAAGAGGTTTGGCATGAAA	CCGGCGCAAGAGGTTTGGCATGAAACTCGAGTTTCATGCCAAACCTCTTGCGTTTTTG
12157	PTGS1	Human	GCCAGTGAATCCCTGTTGTTA	CCGGGCCAGTGAATCCCTGTTGTTACTCGAGTAACAACAGGGATTCACTGGCTTTTTG
12158	PTGS1	Human	CGGCCACATTTATGGAGACAA	CCGGCGGCCACATTTATGGAGACAACTCGAGTTGTCTCCATAAATGTGGCCGTTTTTG
12159	PTGS1	Human	CATGGAGTTCAACCATCTCTA	CCGGCATGGAGTTCAACCATCTCTACTCGAGTAGAGATGGTTGAACTCCATGTTTTTG
12160	PTGS2	Human	GCTGAATTTAACACCCTCTAT	CCGGGCTGAATTTAACACCCTCTATCTCGAGATAGAGGGTGTTAAATTCAGCTTTTTG

12161	PTGS2	Human	GCAGATGAAATACCAGTCTTT	CCGGGCAGATGAAATACCAGTCTTTCTCGAGAAAGACTGGTATTTCATCTGCTTTTTG
12162	PTGS2	Human	CCAGGGCTCAAACATGATGTT	CCGGCCAGGGCTCAAACATGATGTTCTCGAGAACATCATGTTTGAGCCCTGGTTTTTG
12163	PTGS2	Human	CGTTGTGAATAACATTCCCTT	CCGGCGTTGTGAATAACATTCCCTTCTCGAGAAGGGAATGTTATTCACAACGTTTTTG
12164	PTGS2	Human	CCATTCTCCTTGAAAGGACTT	CCGGCCATTCTCCTTGAAAGGACTTCTCGAGAAGTCCTTTCAAGGAGAATGGTTTTTG
